# Donepezil + chromone + melatonin hybrids as promising agents for Alzheimer’s disease therapy

**DOI:** 10.1080/14756366.2018.1545766

**Published:** 2019-02-04

**Authors:** Irene Pachón-Angona, Bernard Refouvelet, Rudolf Andrýs, Helène Martin, Vincent Luzet, Isabel Iriepa, Ignacio Moraleda, Daniel Diez-Iriepa, María-Jesús Oset-Gasque, José Marco-Contelles, Kamil Musilek, Lhassane Ismaili

**Affiliations:** a Neurosciences intégratives et cliniques, Pôle Chimie Organique et Thérapeutique, University Bourgogne Franche-Comté, Besançon, France;; b Faculty of Science, Department of Chemistry, University of Hradec Kralove, Hradec Kralove, Czech Republic;; c PEPITE EA4267, Laboratoire de Toxicologie Cellulaire, University Bourgogne Franche-Comté, Besançon, France;; d Department of Organic Chemistry and Inorganic Chemistry, Alcalà University, Madrid, Spain;; e Institute of Chemical Research Andrés M. del Río, Alcalà University, Madrid, Spain;; f Instituto de Investigación en Neuroquímica, Universidad Complutense de Madrid, Madrid, Spain;; g Department of Biochemistry and Molecular Biology, School of Pharmacy, Plaza de Ramòn y Cajal, Madrid, Spain;; h Laboratory of Medicinal Chemistry (IQOG, CSIC), Madrid, Spain

**Keywords:** Antioxidant, cholinesterases, donepezil, MAO-A, MAO-B, ORAC, Ugi-4MCR

## Abstract

We describe herein the design, multicomponent synthesis and biological studies of new donepezil + chromone + melatonin hybrids as potential agents for Alzheimer’s disease (AD) therapy. We have identified compound **14n** as promising multitarget small molecule showing strong BuChE inhibition (IC_50_ = 11.90 ± 0.05 nM), moderate hAChE (IC_50_ = 1.73 ± 0.34 μM), hMAO A (IC_50_ = 2.78 ± 0.12 μM), and MAO B (IC_50_ = 21.29 ± 3.85 μM) inhibition, while keeping a strong antioxidant power (3.04 TE, ORAC test). Consequently, the results reported here support the development of new multitarget Donepezil + Chromone + Melatonin hybrids, such as compound **14n**, as a potential drug for AD patients cure.

## Graphical Abstract

**Figure:**
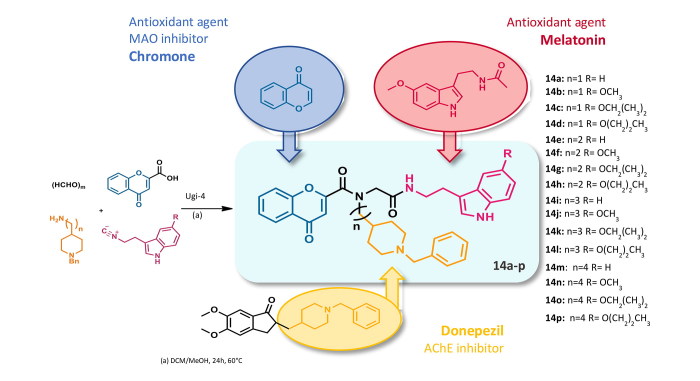

## Introduction

Alzheimer’s disease (AD) remains the most common cause of cognitive impairment in aging population^1^. AD is a multifactorial disorder exhibiting highly interconnected physiopathological processes^2^, leading to a dramatic loss of cholinergic neurons, low level of acetylcholine (ACh), accumulation of intracellular neurofibrillary tangles, abnormal extracellular deposits of amyloid *β* peptide (A*β*), associated to accumulation of biometals (Cu, Fe, Zn) and oxidative stress (OS)^3–5^.

Based on the multifactorial nature of AD and the inadequate therapeutic efficacy of monotarget therapeutic approach, new strategies have been advanced based on the multitarget small molecule (MTSM) approach for the development of new drugs able to bind simultaneously at diverse enzymatic systems or receptors involved in the pathology^6–11^. Accordingly, in our laboratory, we have developed a number of new MTSM as acetylcholinesterase inhibitors (AChEIs) and strong antioxidants, prepared by multicomponent reaction (MCRs)^12–14^.

MCRs have emerged as powerful synthetic methodologies transforming three or more starting materials into new products, in a one-pot procedure^15^ ^,^ ^16^, being the method of choice for introducing rapidly and efficiently chemical diversity^17^. Therefore, they seem well-suited in the search for new MTSM.

**Figure 1.**
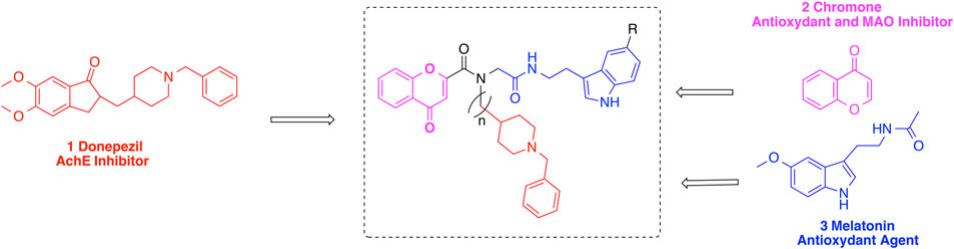
Design of donepezil-chromone-melatonin hybrids.

In this paper, we report the design, synthesis and the biological evaluation of a new family of chromones resulting from the Ugi four-MCR (Ugi-4MCR), by incorporating selected motifs from Donepezil, and Melatonin (Figure 1). Donepezil (**1**) (Figure 1), a ChE inhibitor (ChEI), was the second drug approved by the US FDA for the treatment of mild to moderate AD. Donepezil bears *N*-benzylpiperidine and indanone moieties, and exhibits selectivity for the inhibition of AChE over butyrylcholinesterase (BuChE)^11^.

The Chromone (**2**) (Figure 1) is a privileged scaffold in medicinal chemistry, and the core fragment of several flavonoid derivatives exhibiting a wide range of pharmacological activities^18^, including particularly their ability to inhibit monoamine oxidase enzymes (MAO)^19–21^. It is well known that monoamine oxidase inhibitors (MAOIs) are able to reduce the formation of neurotoxic H_2_O_2_ which increase the formation of reactive oxygen species (ROS) ^22^, and have therapeutic potential for the treatment of AD and Parkinson’s disease^23^ ^,^ ^24^.

The powerful indirect antioxidant functions^25^ of Melatonin (**3**) (Figure 1), and its ability to scavenge different types of ROS in cells^26^, confers to this compound a strong neuroprotective capacity against OS^27^. Melatonin plays likewise a neuroprotective role against A*β* ^28^ and easily crosses the brain-blood barrier.

Lead hybridisation-based synthesis of combinational pharmacophoric groups is a new strategy in drug discovery, of paramount interest and an important starting point for the identification of new chemical entities which are likely to have improved biological activities respect to the standard ligands used as models; in addition, this drug-design approach results in ligands showing diverse selectivity profile, and modes of action minimisingminimising the secondary side effects^29^.

**Scheme 1.**
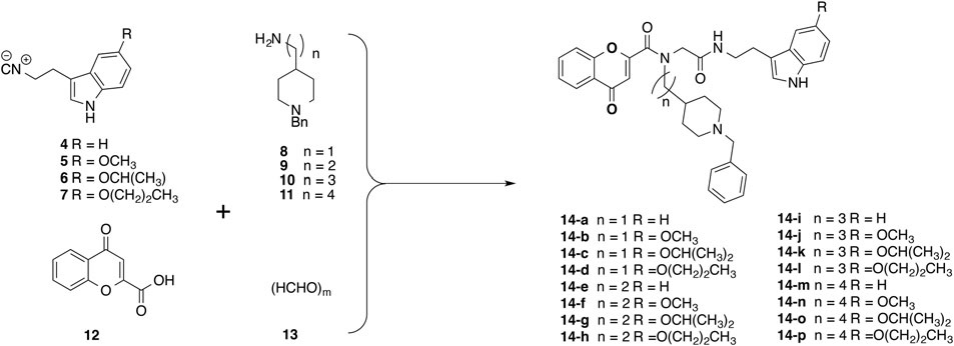
Synthesis of the Donepezil-Chromone_melatonin Hybrids **14a–p.** Conditions: MeOH/CH_2_Cl_2_ (3:1), rt, 24 h.

Considering these precedents, and using a Ugi-4 MCR, we have designed the new MTSM of general structure **I** (Figure 1), synthesisedsynthesised sixteen new hybrids **14a–p** (Scheme 1, Table 1), and evaluated their ChE and MAO inhibition, as well as their antioxidant activity (ORAC-FL test). From these studies, we have identified derivative **14n** (Scheme 1, Table 1) as new and very promising hit-MTSM for the potential AD therapy.

**Table 1.** ORAC-FL values, Inhibition of *Ee*AChE, hACHE, eqBuChE, hMAOA and hMAOB values for **14a–p**, ferulic acid, melatonin, donepezil, tacrine clorgyline, and pargyline.

Ref.	*n*	*R*	ORAC^a^(Eq Trolox)	IC_50_ hMAOA^b^ (μM)	IC_50_ hMAOB^b^ (μM)	IC_50_*Ee*AChE^b^(μM)	IC_50_ eqBuChE^b^(nM)	IC_50_ hAChE^b^(μM)	IC_50_ hBuChE^b^(nM)
^14^a	1	H	6.28 ± 0.12	2,00 ± 0,13	*–*^c^	–^d^	496.02 ± 7.07	–^d^	1167.30 ± 19.05
^14^b	1	OCH_3_	6.24 ± 0.05	–^c^	–^c^	–^d^	636.23 ± 11.64	–^d^	–^d^
^14^c	1	OCH(CH_3_)_2_	6.11 ± 0.07	–^c^	–^c^	–^d^	245.73 ± 9.91	–^d^	–^d^
^14^d	1	O(CH_2_)_2_CH_3_	6.76 ± 0.09	–^c^	–^a^	–^d^	46.89 ± 3.41	–^d^	–^d^
^14^e	2	H	6.52 ± 0.37	–^c^	–^c^	1.43 ± 0.05	29.59 ± 2.38	2.62 ± 0.53	33.30 ± 0.55
^14^f	2	OCH_3_	6.13 ± 0.09	–^c^	80.59 ± 13.88	0.69 ± 0,03	26.03 ± 2.37	0.36 ± 0.16	271.75 ± 1.75
^14^g	2	OCH(CH_3_)_2_	5.05 ± 0.15	–^c^	–^c^	1.05 ± 0.06	6.29 ± 0.73	2.00 ± 0.07	25.00 ± 0.20
^14^h	2	O(CH_2_)_2_CH_3_	6.07 ± 0.02	–^c^	–^c^	0.85 ± 0.02	6.76 ± 0.49	2.04 ± 0.66	63.90 ± 2.55
^14^i	3	H	6.92 ± 0.32	–^c^	–^c^	0.26 ± 0.01	50.11 ± 5.19	–^d^	–^d^
^14^j	3	OCH_3_	3.31 ± 0.10	–^c^	–^c^	0.27 ± 0.00	37.06 ± 0.46	–^d^	–^d^
^14^k	3	OCH(CH_3_)_2_	2.27 ± 0.37	2.99 ± 0.16	15.17 ± 2.33	0.36 ± 0.01	18.21 ± 0.18	–^b^	34.90 ± 0.85
^14^l	3	O(CH_2_)_2_CH_3_	2.44 ± 0.16	–^c^	–^c^	0.38 ± 0.01	9.03 ± 0.18	–^d^	–^d^
^14^m	4	H	2.97 ± 0.16	–^c^	–^c^	0.06 ± 0.00	355.33 ± 8.41	–^d^	–^d^
^14^n	4	OCH_3_	3.04 ± 0.36	2.78 ± 0.12	21.29 ± 3.85	0.48 ± 0.01	43.38 ± 1.37	1.73 ± 0.34	11.90 ± 0.05
^14^o	4	OCH(CH_3_)_2_	2.63 ± 0.12	–^c^	–^c^	0.09 ± 0.00	46.62 ± 2.76	–^d^	–^d^
^14^p	4	O(CH_2_)_2_CH_3_	2.67 ± 0.15	1.39 ± 0.11	*–*^c^	0.08 ± 0.00	61.33 ± 5.48	2.10 ± 0.51	80.35 ± 12.35
Ferulic acid	–	–	3.74 ± 0.22	*–*^e^	*–*^e^	*–*^e^	*–*^e^	*–*^e^	*–*^e^
Melatonin	–	–	2.45 ± 0.09	*–*^e^	*–*^e^	*–*^e^	*–*^e^	*–*^e^	*–*^e^
Donepezil	–	–	*–*^e^	*–*^e^	*–*^e^	0.02 ± 0.00	840 ± 20	0.023 ± 0.01	7420 ± 390
Tacrine	–	–	0.2 ± 0.1	*–*^e^	*–*^e^	0.03 ± 0.01	5.1 ± 0.1	0.424 ± 21^37^	45.8 ± 3.0^37^
Clorgyline	–	–	*–*^e^	0.05 ± 0.00	*–*^e^	*–*^e^	*–*^e^	*–*^e^	*–*^e^
Pargyline	–	–	*–*^e^	*–*^e^	0.08 ± 0.01	*–*^e^	*–*^e^	*–*^e^	*–*^e^

a Data are expressed as Trolox equivalents and are the mean (*n* = 3) ± SD.

b Each IC_50_ value is the mean  ±  SEM of at least three different experiments.

c Enzymatic inhibition (%) <40% at 10 μM;.

d Enzymatic inhibition (%) <50% at 10 μM;.

e not determined.

## Methods

### Chemistry

#### General procedure for the ugi-4MCR synthesis of compounds

A solution of the corresponding *N*-benzylpiperidines **8**–**11** (1.0 mmol) and paraformaldehyde (**13**) (1.0 mmol) in MeOH/CH_2_Cl_2_ (7 ml, 3:1, v/v) was stirred for 1 h at room temperature (rt). Chromone-2-carboxylic acid (**12**) (1.0 mmol) and the appropriate isocyanide **4**–**7** (1.0 mmol) were then added. The reaction was stirred 24 h at rt, and when complete (tlc control), the crude product was isolated and purified by flash column chromatography to afford the corresponding hybrids **14a–p** (see Supplementary Material).

### Biological evaluation

#### Oxygen radical absorbance capacity assay

The antioxidant activity of hybrids **14a–p** was carried out by the ORAC-FL using fluorescein as a fluorescent probe. Briefly, fluorescein and antioxidant were incubated in a black 96-well microplate (Nunc) for 15 min at 37 °C. The 2,2’-azobis(amidinopropane) dihydrochloride was then added quickly using the built-in injector of Varioskan Flash plate reader (Thermo Scientific). The fluorescence was measured at 485 nm (excitation wavelength) and 535 nm (emission wavelength) each min for 1 h^30^ ^,^ ^31^.

#### MAO activity assay

The reaction mixture contains 2.5 μg/mL MAO-A or 6.25 μg/mL MAO-B enzyme and inhibitor in final concentrations of 1, 5, 8, 10, 15, 30, 50, and 80 μM in 50 mM potassium phosphate buffer with 20% (v/v) glycerol (pH 7.5). The mixture was pre-incubated at 37 °C for 5 min and subsequently, substrate kynuramine was added to the final concentration of 60 μM in case of MAO-A and 30 μM in case of MAO-B. The final volume of reaction mixture was 0.1 ml. The whole reaction mixture was incubated at 37 °C for 30 min. The reaction was stopped by the addition of 200 μl acetonitrile:methanol mixture (ratio 1:1) and cooling down to 0 °C. The sample was then centrifuged (16.500 g) for 10 min.

#### Metabolite determination

The deamination product of kynuramine formed during the enzymatic reaction 4-hydroxyquinoline (4-HQ) was determined by HPLC–MS on a 2.1 mm × 50 mm, 1.8 μm Zorbax RRHD Eclipse plus C18 column (Agilent), by using an 6470 Series Triple Quadrupole mass spectrometer (Agilent) (electrospray ionisation - positive ion mode). Three m/z MRM transitions were followed for kynuramine (165.1= >30.2, 165.1= >118.0, 165.1= >136.0) and 4-HQ (146.1= >51.1, 146.1= >77.0, 146.1= >91.0). Eluents: (A) 0.1% formic acid in water; (B) 0.1% formic acid in acetonitrile. IC_50_ of individual compounds were determined using GraphPad Prism 6.0, San Diego, CA.

#### ChE activity assay

Inhibition of *Ee*AChE and eqBuChE of hybrids **14a–p** was carried out with *Ee*AChE (type VI-S, purified from *Electrophorus electricus*) and eqBuChE (purified from equine serum) using Ellman’s protocol^32^. Briefly, the reaction was performed in a final volume of 3 ml of a phosphate-buffered solution (0.1 M) at pH = 8.0 containing 5,5’-dithiobis-2-nitrobenzoic acid (DTNB) (2.625 μL, 0.35 mM), *Ee*AChE (29 μl, 0.035 U/mL) or eqBuChE (60 μl, 0.05 U/mL), Bovine Albumin Serum phosphate-buffered solution (40 μL 1% w/v), and tested compound (3 µL). After 10 min of incubation, the substrate, AcTCh) iodide or butyrylthiocholine (BuTCh) iodide, was added, and the reaction was incubated for an additional 15 min. The absorbances were measured in a spectrometric plate reader at 412 nm and the IC_50_ were determined using GraphPad Prism 5, San Diego, CA.

To investigate the inhibitory capacity of the compounds on hAChE and hBuChE, Ellman's spectrophotometric method^32^ was used with minor modifications^33^, using recombinant expressed in HEK 293 hAChE (Sigma Aldrich, Madrid, Spain) and hBuChE from human serum (Sigma Aldrich, Madrid, Spain). The reaction was carried out in a 48-well plate at 37 °C on a final volume of 500 μL of a phosphate buffer solution (TPS 0.1 M pH 8), each well containing 200 μL of 0.875 mM of DTNB (5,5′-dithiobis -2-nitrobenzoic acid in PBS 1×) and 5 μL hAChE (CSU 3.6 U/mL) or 5 μL hBuChE (CSU 5 U/mL) for a final concentration of 0.036 U/mL or 0.05 U/mL in each well. The reaction was started by adding acetylthiocholine (AcTCh) at 0.35 mM (Cf in the well) for hAChE, and butyrylthiocholine (BuTCh) at 1 mM (Cf in the well). The enzymatic reaction was monitored at 410 nm of absorbance during 5 min for hAChE and 8 min for hBuChE in a Biotek Power Wave XS spectrophotometer plate reader (kinetic measure each 15 s). Inhibition curves were performed by pre-incubation of this mixture with at least five concentrations of each compound (10 nM–100 μM in case of hAChE, and 10 pM-75 μM in case of hBuChE) for 10 min. A control sample (without compound) and a tacrine dilution at IC_50_ concentration (500 nM for hAChE and 30 nM for hBuChE) were always included to determine 100% of the enzymatic activity in each assay. After this pre-incubation period, the corresponding substrate was added and put in the spectrophotometer during 5 or 8 min for the colour change detection of the enzymatic reaction.

### Molecular docking of compound 14n into hAChE and hBuChE

The structure of compound **14n** was built as its hydrochloride using standard bond lengths and bond angles with Discovery Studio, version 2.1, software package, San Diego, CA. The molecular geometry of **14n** was energy-minimisedminimised with the CHARMm force field^34^ using the adopted-based Newton–Raphson algorithm, considering this structure fully optimisedoptimised when the energy changes between iterations were less than kcal(mol-Å)^−1^ ^35^. The ligand was set up for docking with the help of AutoDockTools (ADT; version 1.5.4) to define the torsional degrees of freedom to be considered during the docking process. All the acyclic dihedral angles in the ligand were allowed to rotate freely.

The enzyme structure used for the calculations was the human AChE (Protein Data Band code 1B41). For docking purposes, the initial protein was prepared by removing all water molecules, heteroatoms, any co-crystallised solvent, and the ligand. Proper bonds, bond orders, hybridisationhybridisation, and charges were assigned using protein model tool in Discovery Studio, version 2.1, software package, San Diego, CA. CHARMm force field was applied using the receptor-ligand interactions tool in Discovery Studio, version 2.1, software package. ADT was used to add hydrogens and partial charges for proteins and ligands using Gasteiger charges. To give flexibility to the binding site, residues lining the site were allowed to move. These residues were: Trp286, Tyr124, Tyr337, Tyr341, Tyr72, Asp74, Thr75, Trp86. The grid box was built with a resolution of 1 Å and 60 × 60 × 72 points and it was positioned at the middle of the protein (*x* = 116.546; *y* = 110.33; *z* = −134.181). The docking box displayed using ADT constituted a large region to include whole protein target (“blind docking”).

For hBuChE (4BDS) docking calculations were performed following a similar protocol described before for hAChE. Blind dockings were performed by considering a cube of 75 A° with grid points spacing of 1 Å positioned at the middle of the protein (*x* = 137.985; *y* = 122.725; *z* = 38.78).

Docking calculations were performed with the programme Autodock Vina^36^. Default parameters were used except num_modes, which was set to 40.

### Molecular docking of compound 14n into human MAO a/B

Compound **14n** was built as non-protonated amine following the procedure described before for cholinesterases. The crystal structures of human MAO-A in complex with harmine (PDB ID 2Z5X) and human MAO-B crystallised with safinamide (PDB ID 2V5Z) were retrieved from the Protein Data Bank. The enzymes structures were prepared for docking. First, in the PDB crystallographic structures, any co-crystallised solvent and the ligand were removed. Then, proper bonds, bond orders, hybridisationhybridisation, and charges were assigned using protein model tool in Discovery Studio, version 2.1, software package, San Diego, CA. CHARMm force field^34^ was applied using the receptor-ligand interactions tool in Discovery Studio, version 2.1, software package, San Diego, CA. During the protein set up, six water molecules located around the FAD cofactor were considered in the docking experiments because they are essential for stabilisingstabilising the complexes as also reported in the literature. Finally, atoms of the FAD cofactor were defined in their oxidisedoxidised state.

The ADT programme was used to generate the docking input files. For MAO-A, the grid centre coordinates were *x* = 53.065, *y* = 161.664, *z* = 26.000 and the size coordinates were *x* = 24, *y* = 34, *z* = 38 with grid points separated 1 Å; for MAO-B, the grid centre coordinates were *x* = 53.065, *y* = 161.664, *z* = 29.046 and the size coordinates were *x* = 24, *y* = 34, *z* = 36 with grid points separated 1 Å.

Docking calculations were performed following the same protocol described before for hAChE.

According to Vina best-scored poses, the most stable complex configurations were considered. The docked ligand output files were viewed and hydrogen bond interactions, van der Waals interactions as well as electrostatic interaction were evaluated using Discovery Studio, San Diego, CA.

## Results and discussion

### Chemistry

The synthesis of the target compounds **14a–p** was carried out as shown in Scheme 1. The *one-pot* Ugi-4MCR^37^, with isocyanides **4**–**7**, (prepared in two steps respectively from tryptamine, 5-methoxytryptamine, *iso*propyloxytryptamine^38^ and propyloxytryptamine^38^), formaldehyde (**13**), the appropriate commercial amines **8**, **9**, 3–(1-benzylpiperidin-4-yl)propan-1-amine (**10**)^39^, 4–(1-benzylpiperidin-4-yl)butan-1-amine (**11**)^39^ ^,^ ^40^, and 4-oxo-4*H*-chromene-2-carboxylic acid (**12**), in MeOH/CH_2_Cl_2_ (3:1) at rt for 24 h, afforded the desired compounds **14a–p** from low to moderate yields. All new compounds showed analytical and spectroscopic data in good agreement with their structure (see Supplementary Material).

### Biological evaluation

The *in vitro* evaluation of compounds **14** started with the analysis of their antioxidant activity, continued with the inhibition of MAO-A and MAO-B and ended with the evaluation of ChEs inhibition.

### Antioxidant activity

The antioxidant activity of hybrids **14a–p** was determined by the ORAC-FL method^30^ ^,^ ^41^. Results are expressed in relation to radical scavenging properties of Trolox yielding the Trolox equivalents (TE) unit. Ferulic acid and Melatonin were used as positive references molecules showing ORAC values of 3.74^12^ and 2.45^12^, respectively.

**Figure 2.**
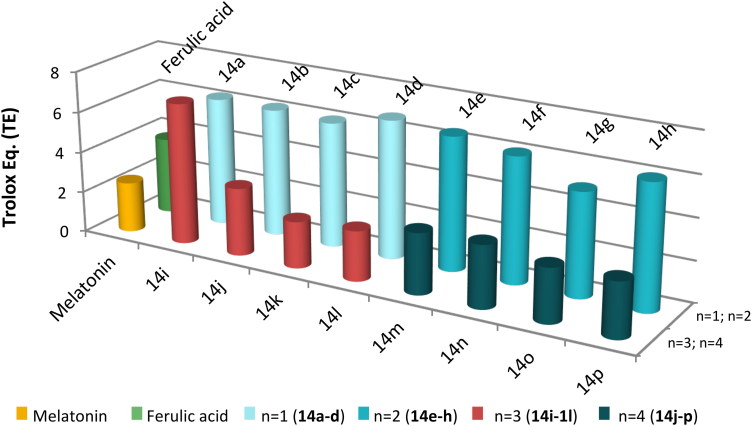
Antioxidant power of compounds **14a–p** compared to melatonin and ferulic acid.

As shown in Table 1 and Figure 2, all compounds presented strong radical scavenging properties with TE values ranging from 2.27 (**14k**) to 6.92 (**14i**). In general, compounds with a length in the linker of *n* = 1, 2 are more potent antioxidants than Ferulic acid; however, and except for hybrid **14i,** those with *n* = 3, 4 are fewer antioxidants than Ferulic acid, but remain comparable to Melatonin.

### *In vitro* inhibition of human MAOs

Clorogyline, an irreversible and selective MAOAI and pargyline, an irreversible and selective MAOBI, were used as references. As shown in Table 1, compounds **14a, 14k, 14n,** and **14p** can effectively inhibit MAO-A at micromolar range with IC_50_ values equal to 1.99, 2.99, 2.79, and 1.39 μM, respectively, and therefore they are less active than clorogyline (IC_50_ = 0.05 μM). Hybrids **14a** and **14p** are selective MAO-AI, while compounds **14k** and **14n** also inhibit MAO-B (IC_50_ = 15.2 and 21.3 μM), respectively, and are less potent compared to Pargyline (IC_50_ = 0.08 μM). Finally, compound **14f** (IC_50_ = 80.59 μM) is a modest and selective MAOBI.

### *In vitro* inhibition of eeAChE and eqBuChE

To complete the multitarget biological analysis, we analysedanalysed the *Ee*AChE and eqBuChE inhibition, using Donepezil as a reference agent, and the Ellman protocol^32^ for the determination of enzyme inhibition. As shown in Table 1, the target compounds **14a–p** are generally more active against eqBuChE than *Ee*AChE. The most effective eqBuChEI are compounds **14g** (IC_50_ = 6.76 nM) and **14h** (IC_50_ = 6.29 nM), what means that molecules **14g** and **14 h** are 133-fold more potent than Donepezil and have an inhibitory potency similar to tacrine. It is worth noting that excluding hybrids **14a–c** and **14m**, the other twelve compounds are potent BuChEI, in the nanomolar range, showing IC_50_ (nM) values from 6.3 (**14g** and **14h)** to 50.1 (**14i**).

Regarding the structure-activity relationship, and for the same substituent, the most potent inhibitors are those bearing a length in the linker of *n* = 2, and the less potent for *n* = 3, 1 and 4, respectively. However, for the same linker length, the most potent compounds are always those bearing propoxy or isopropoxy substituents at the indole ring, such as hybrids **14c,d,g,h,k,l,o,p.**

For the *Ee*AChE inhibition, the less potent inhibitors **14a–d** are those with *n* = 1 displaying a percentage of inhibition lower than 50% at 10 μM concentration, while the three stronger *Ee*AChEI, showing IC_50_ values ranging from 58.2 (**14m**) to 88.5 nM (**14o**) correlate with *n* = 4 as length in the linker. Compounds **14i–l** with *n* = 3 are more potent than hybrids **14e–h** with *n* = 2, confirming thus that the inhibition of *Ee*AChE increases with the length in the linker. Finally, it is worth of note that compounds **14o–p** are the stronger inhibitors for both *Ee*AChE and eqBuChE.

### In vitro inhibition of hChE

Based on these results, we have selected the most balanced compounds **14a**, **14e–h**, **14k**, **14n,** and **14p**, in terms of the antioxidant properties, MAO-A/B and *Ee*AChE/eqBuChE inhibitory activities, for the study of the human cholinesterases (hChEs) inhibition. As shown in Table 1, the tested compounds showed higher inhibitory potency against hBuChE compared to hAChE. The inhibitory activity on hBuChE ranges from 11.90 nM (**14n**) to 1167 nM (**14a**), and six of the eight tested compounds (**14e,g,h,k,n,** and **p**) have IC_50_ values under 85 nM; moreover, hybrids **14a** and **14k** are totally selective hBuChEI. The most potent hBuChEI is compound **14n,** 70-fold more active than Donepezil and only 2-fold less active than Tacrine. With respect to hAChE inhibition, the tested molecules showed IC_50_ values in micromolar range, between 2.62 μM for **14e** and 0.36 μM for the most active compound **14f**, 15-fold less active than Donepezil.

### Molecular docking studies

Docking studies were carried out in order to discover the binding modes and the interactions of compound **14n** with hAChE, hBuChE, hMAO-A, and hMAO-B. Docking simulations and visualisationsvisualisations were performed using AutoDock Vina^36^ and Discovery Studio, San Diego, CA.

### AChE molecular modelling studies

The 3 D structure of hAChE was retrieved from the PDB (coded as 1B41). Multiple AChE X-ray structures have revealed a large mobility of some residues to accommodate ligands. Therefore, the flexibility of eight side chains have been taken into account in docking strategies by allowing side chain flexibility during the docking search as performed by software Autodock Vina^36^

**Figure 3.**
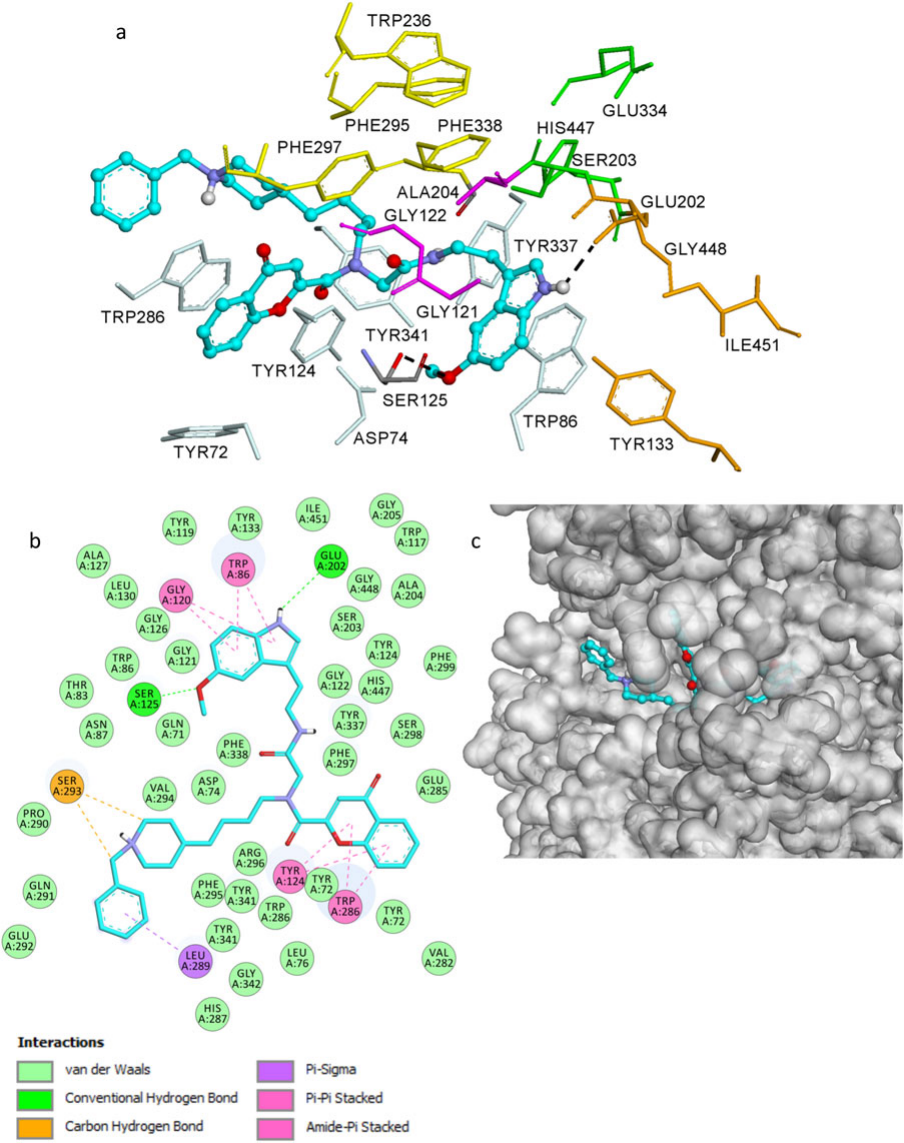
Docked pose of inhibitor **14n** at the active site of hAChE. (a) Compound **14n** is rendered as ball and sticks (carbon atoms in blue) and the side chain conformations of the mobile residues are illustrated in light blue. The catalytic triad (CT) is Coloured in green, the oxyanion hole (OH) in magenta, the anionic sub site (AS) in orange, except Trp86, the acyl binding pocket (ABP) in yellow and PAS in light blue. (b) 2D-representation of interactions established by **14n** with hAChE. (c) 3D surface representation of hAChE (white) with compound **14n** (blue).

As shown in Figures 3(a,c), compound **14n** located in the entire enzymatic gorge. Docking simulations showed a clear preference to accommodate the chromone moiety within the PAS while the binding pocket forming the CAS is embedded by the methoxyindole moiety with the flexible linker lying in the middle of the gorge between CAS and PAS.

In the complex, the indole moiety is pointed toward the catalytic triad residues, His447, Ser203, and Glu334 but it does not interact via hydrogen bonding or hydrophobic interactions with any residue of the catalytic triad. It was found that the indole moiety of the ligand is forming π–π interaction and amide-π stacked interaction with Trp86 and with Gly120, respectively. In addition, the NH and methoxy groups are involved in hydrogen bonds with Glu202 and Ser125, respectively.

The chromone moiety is located in the pocket forming PAS interacting with Tyr124 and Trp286 via face-to-face π–π stacking interactions. In this situation, the *N*-benzylpiperidinium moiety is turned away from the PAS interacting with Leu289 and Ser293 (Figure 3(b)).

### BuChE molecular modelling studies

**Figure 4.**
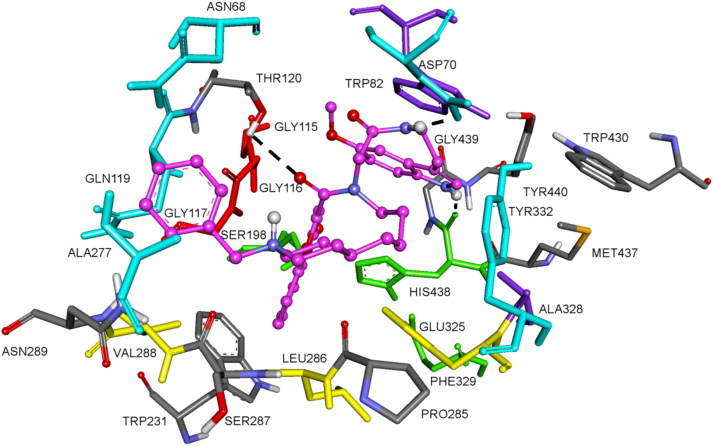
Proposed binding mode for compound **14n** inside gorge cavity of hBuChE. Compound **14n** is Coloured pink. Different subsites of the active site were Coloured: catalytically anionic site (CAS) in green, oxyanion hole (OH) in red, choline binding site in violet (CBS), acyl binding pocket (ABP) in yellow, and peripheral site (PAS) in blue.

Compound 1**4n** was modelled into the structure of hBuChE (PDB: 4BDS). The ligand fits into the active site of hBuChE mainly through π–π and hydrogen bonds interactions, which are found to be essential for binding. The binding mode of compound **14n** at the active site of hBuChE is illustrated in Figure 4. The docking results showed that in the lowest energy binding orientation of the ligand, the chromone moiety formed π–π T-shaped interactions with Phe329 (in the anionic site of the enzyme) and Trp231. The indole moiety binds in the CAS region of the enzyme, establishing π–π stacking interactions with Trp82 and His438 of the catalytic triad.

**Figure 5.**
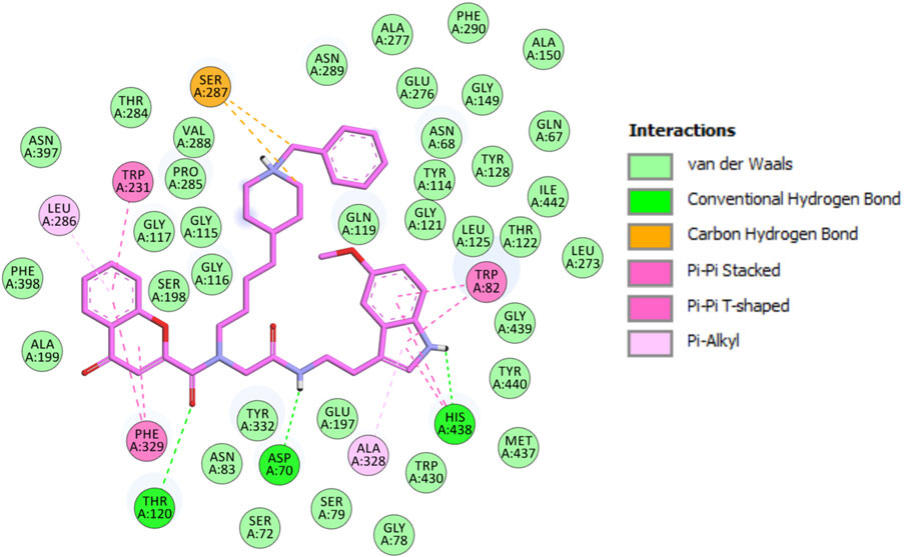
2D Schematic representation of different interactions of compound **14n** with hBuChE.

Besides, the NH group forms a hydrogen bond interaction with His438. The ligand displays additional hydrogen bond interactions between the C=O group of the di-substituted amide with Thr120 and the NH group of the mono-substituted amide with Asp70 in the PAS (Figure 5).

Besides the key ligand-enzyme interactions described above, the difference in the AChE and BuChE inhibitory activities may be mainly explained by the inverted orientation adopted by the ligand on both enzymes. It is also noteworthy that for the compound **14n** with three branches, both ending in chromone and indole rings play a key role in the cholinesterase inhibitory activity. The alkyl- *N*-benzylpiperidinium chain placed outside the active site can be important in helping anchor the ligand to the protein.

### MAO molecular modelling studies

In order to explore the nature of the ligand-receptor interactions, compound **14n** was docked to the active site of both MAO-A and MAO-B isoforms using the programme Autodock Vina^36^.

Docking simulations were run on the human model of the MAO-A and MAO-B isoforms. The 3 D structures for hMAOs were retrieved from the PDB (PDB ID: 2Z5X for hMAO A and PDB ID: 2V5Z for hMAO B).

Results from several studies have shown that it must be the neutral amine that reaches the active site of MAO A and MAO B that allows the chemistry^42–45^. Based on this fact, the neutral form of **14n** was docked into MAOs enzymes despite that at physiological pH, most of the piperidine rings would be in the protonated, positively charged form.

As reported in our previous papers^46–48^, six water molecules were considered as integral components of the protein structure in the docking simulations. These water molecules are labelled as w72, w193, w11, w23, w15, and w53 according to the numbering reported in the hMAO B crystallographic structure (PDB ID: 1S3E) and they are located near the flavin adenine dinucleotide (FAD) cofactor.

**Figure 6.**
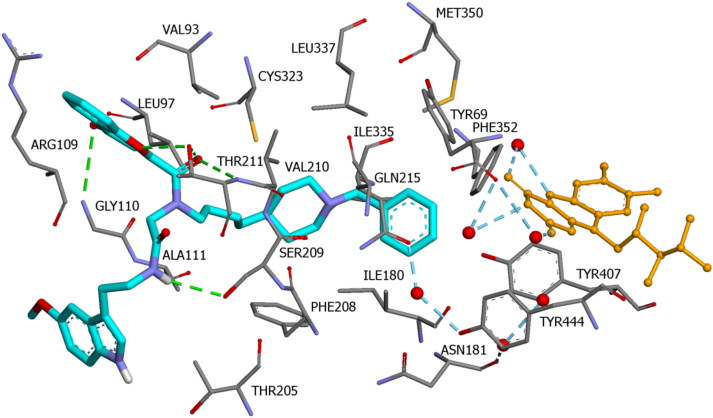
Binding mode of compound **14n** in the active region of hMAO A. Amino acids residues of binding site are colour-coded. The FAD cofactor and the six water molecules represented as an integral part of the MAO-A structure model are rendered as orange balls and sticks and red balls, respectively. Green and blue dashed lines represented hydrogen bond interactions.

**Figure 7.**
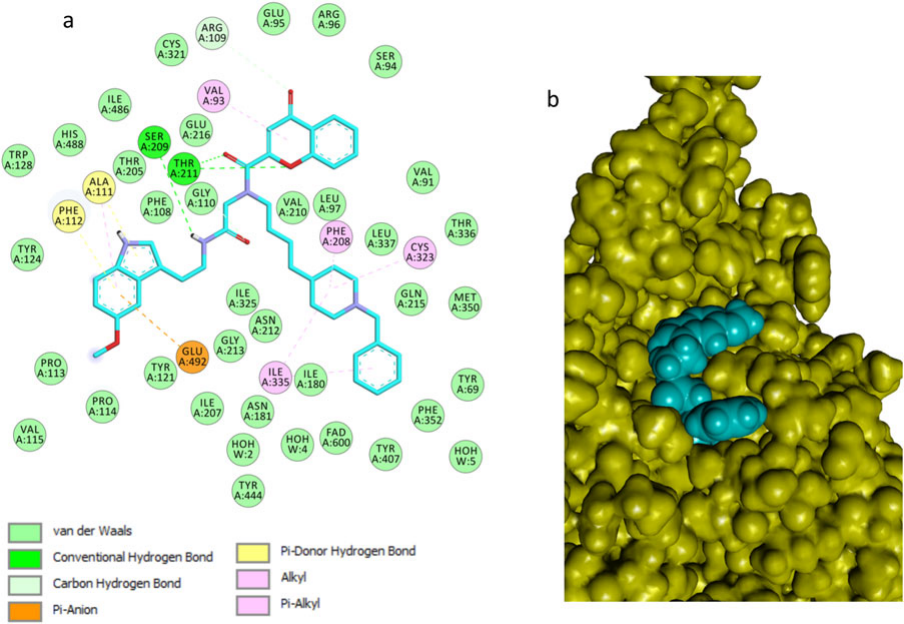
Binding mode of compound **14n** at the binding site of hMAO A. (a) 2D ligand-receptor interaction diagram. (b) Complex of compound **14n** (blue) and hMAO A (yellow).

Figure 6 illustrates the binding mode of **14n** into the hMAO A binding cavity (binding energy: -8.3 kcal/mol). Visual inspection of the pose of compound **14n** into the MAO-A binding site revealed that the phenyl ring placed in the “aromatic cage” framed by Tyr69, Phe352, Tyr407, Tyr444 side chains, as well as the isoalloxazine FAD ring. The aromatic ring is oriented to establish π–alkyl interactions with Ile180 residue. The piperidine ring is located in a hydrophobic core delimited by residues Phe208, Ileu335, and Leu337 (Figure 7(a)).

In the entrance cavity, the chromone moiety is located in a hydrophobic core delimited by residues Val93, Leu97, Gly110, Ala111, and Thr211 (Figure 6). In this orientation, four hydrogen bonds are established, two of them involving the C=O group close to the chromone ring and Thr211; the other two involving the oxygen and the C=O group of the ring and Thr211 and Gly110. Moreover, the NH group of the amide moiety is also able to form a hydrogen bond with Ser209 side chain (Figure 7(a)).

The indole heterocyclic ring did not portray any significant interactions with the MAO-A active site, but it was well accommodated outside in a cleft made up Ala111, Phe112, Pro113, Pro114, Val115, Tyr124, and Glu492 (Figures 7(a,b)).

To rationaliserationalise the selectivity of MAO A/B, docking studies of compound **14n** into the MAO B were done. The six structural water molecules selected for hMAO A were also included in the study.

**Figure 8.**
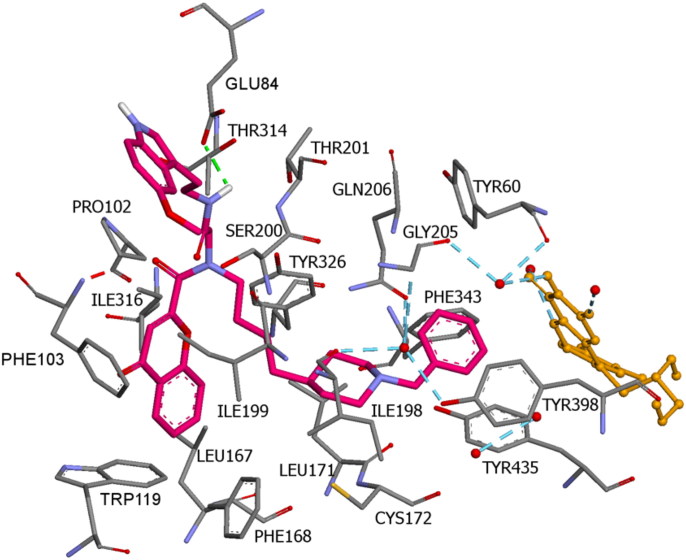
Compound **14n** in the active region of hMAO B. Amino acids residues of binding site are colour-coded. The FAD cofactor and the six water molecules represented as an integral part of the MAO B structure model are rendered as orange balls and sticks and red balls, respectively. Hydrogen bonds are shown with green and blue dashed lines.

**Figure 9.**
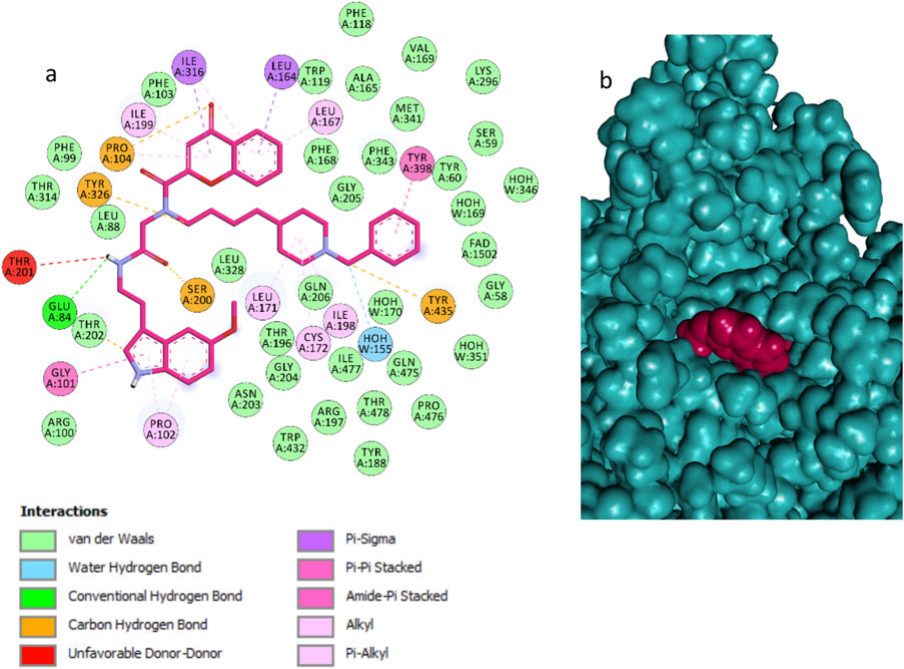
Binding mode of compound **14n** in the active region of hMAO B. Aminoacids residues of binding sire are colour-coded. The FAD cofactor and the six water molecules represented as an integral part of the MAO-A structure model are rendered as orange ball and sticks and red balls, respectively. Green and blue dashed lines represented hydrogen bond interactions.

Docking of compound **14n** into the MAO B binding site revealed that this inhibitor also crosses both cavities, presenting the piperidine nucleus located between the “entrance” and “catalytic” cavities, separated by the residues Ile199 and Tyr326 (Figure 8; binding energy: -7.6 kcal/mol). The phenyl ring is oriented toward the bottom of the substrate cavity, interacting with the FAD cofactor as well as Tyr60, Phe343, Gly205, and Tyr398, through van der Waals interactions and π–π interactions. In addition, the chromone moiety was hosted in the large entrance cavity made up by Phe103, Pro104, Trp119, Leu164, Leu167, Phe168, Ile199, and Ileu316. Moreover, the NH amide group formed a hydrogen bond with Glu84. Finally, the indole ring is oriented to the mouth of the entrance cavity in a hydrophobic sub-pocket, which is defined by Pro102, Thr202, Gly101, Arg100, Thr196, and Asn203 (Figure 9(a)).

The different trends regarding the inhibitory activity of **14n** in MAO A and MAO B can be mainly ascribed to the residues that define the bottleneck in the gorge that leads to the ligand binding site. The replacement of Ile199 in MAO B by Phe208 in MAO A pushes slightly the ligand away from the FAD, this cause that it binds more tightly to MAO A and exhibits a greater number of favorable interactions.

In conclusion, the docking simulations have characterisedcharacterised the binding of compound **14n** at the ChE and MAO enzymes. It seems clear that the chains ending in pyrrole and chromone rings are crucial for binding to the active site of the ChEs. However, for MAOs binding is the *N*-benzylpiperidine chain.

## Conclusion

In this work, we have described the straightforward Ugi-4MCR synthesis and the biological evaluation of the Donepezil + chromone + melatonin hybrids **14a–p** (Scheme 1, Table 1), as antioxidants, MAOIs, and ChEIs for the potential treatment of AD. From these results, we have identified *N*-(4–(1-benzylpiperidin-4-yl)butyl)-*N*-(2-((2–(5-methoxy-1*H*-indol-3-yl)ethyl)amino)-2-oxoethyl)-4-oxo-4*H*-chromene-2-carboxamide (**14n**), as suitable MTSM for further development. Hybrid **14n** shows strong hBuChE (IC_50_ = 11.90 ± 0.05 nM), moderate hAChE, hMAO A, and MAO-B (IC_50_ = 1.73 ± 0.34 μM, IC_50_ = 2.78 ± 0.12 μM, IC_50_ = 21.29 ± 3.85 μM) inhibition, respectively, while keeping a strong antioxidant effect (3.04 TE).

The inhibition of hybrid **14n** towards BuChE is worth of note, as in patients with moderate to severe forms of AD, AChE activity is decreased, and BuChE, elevated^49^, suggesting that ACh hydrolysis in cholinergic synapses may largely occur *via* BuChE catalysis^50^ and that the specific inhibition of BuChE may be important in raising ACh levels, in order to improve the cognition impairment^51^.

To sum up, the present results support the development of new MTSM based on Donepezil + chromone-melatonin hybrids for the potential treatment of AD. This is being investigated in our laboratories and will be reported in due course.

## Supplementary Material

Supplemental Material
