# Supplementary material for: Donepezil + chromone + melatonin hybrids as promising agents for Alzheimer’s disease therapy
Source: J Enzyme Inhib Med Chem. 2019 Feb 4;34(1):479–89. doi: 10.1080/14756366.2018.1545766 (PMC6366423; doi:10.1080/14756366.2018.1545766)
Supplement: Supplemental Material [file IENZ_A_1545766_SM2711.pdf]

Donepezil+Chromone+Melatonin Hybrids as Promising Agents for Alzheimer's Disease Therapy

Supporting information

Synthesis of amines (10 and 11)........S**1**

*Synthesis of **10** and **11***........S1-S2

General procedure A - Synthesis of isocyanides (4-7)........S**3**

General procedure A.1........S3

General procedure A.2........S3

Synthesis of N-formamides (4a-7a)........S4-S5

Synthesis of isocyanides (4-7)........S6-S7

General procedure B - Synthesis of Ugi adducts (14a-p)........S**8**

*Synthesis of **14a-p***........S8-S23

References........S**24**

Synthesis

Amines synthesis (10 and 11):

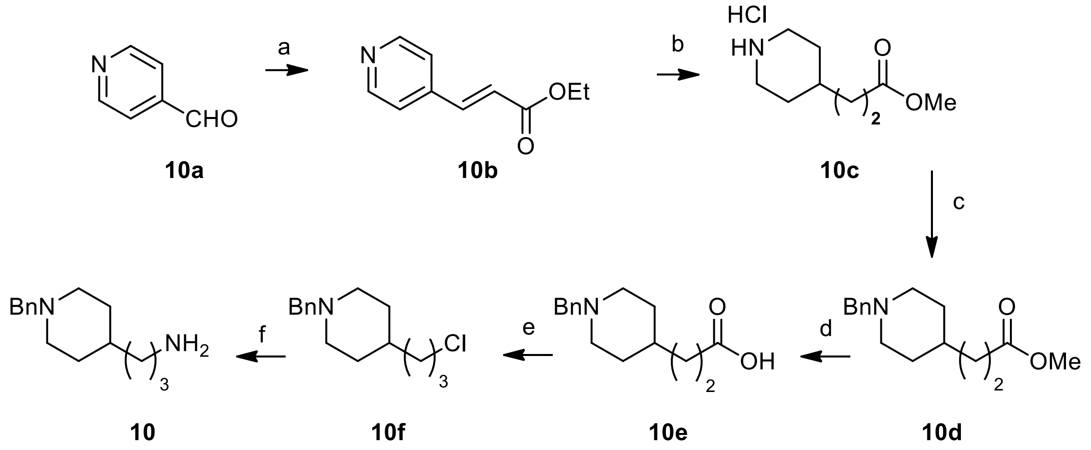

Scheme for 10 synthesis. ***Reagents and conditions***[1]**:** (a) $(EtO)_2P(O)CH_2CO_2Et$, THF, K_2CO_3, reflux, 1h; (b) (i) H_2, Pd/C 10%, PtO_2, 4N HCl in dioxane, EtOH, room temp, O.N. (ii) MeOH; (c) BnBr, Et_3N, CH_2Cl_2, O.N.; (d) $LiAlH_4$, dry THF, reflux, 2h; (e) $SOCl_2$, CH_2Cl_2, reflux, 3h; (f) NH_3/MeOH 7M, 5mbarr, 5h.

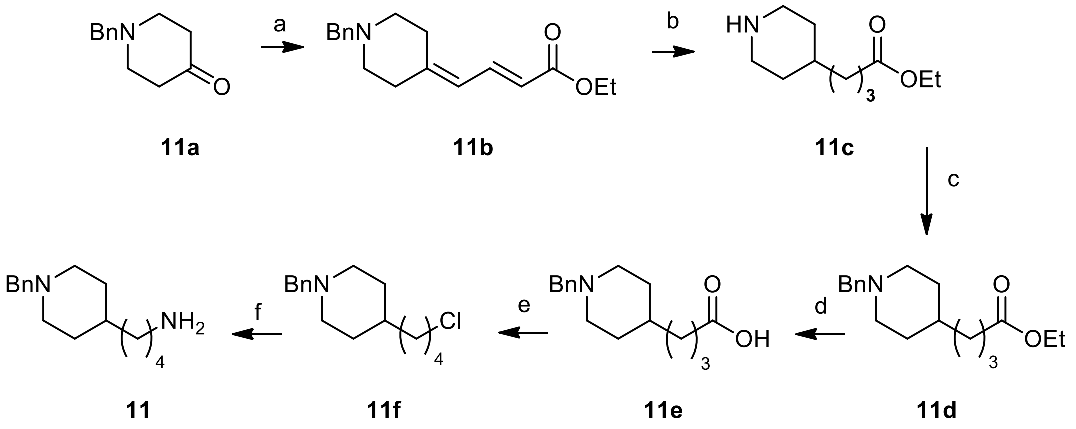

Scheme for 11 synthesis. ***Reagents and conditions***[1]***:*** (a) $(EtO)_2P(O)CH_2CH{=}CHCO_2Et$, EtOH, NaH, Reflux, 1h; (b) H_2, Pd/C 10%, 40mbarr, EtOH, RT O.N.; (c) BnBr, Et_3N, CH_2Cl_2, O.N.; (d) $LiAlH_4$, dry THF, reflux, 2h; (e) $SOCl_2$, CH_2Cl_2, reflux, 3h; (f) NH_3/MeOH 7M, 5mbarr, 5h.

4-(1-benzylpiperidin-4-yl)propan-1-amine (10).

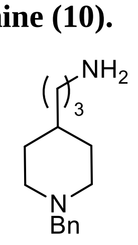

10 $C_{15}H_{24}N_2$

MW: 232.194 g/mol

10 was synthesized starting from *1-benzyl-4-(3-chlorobutyl)piperidine* **10f** which, in turn, was prepared in five steps as described by Choi et al[1] and Bolea et al [2]. **10f (1 Eq., 8 mmol, 2014.00 mg)** was dissolved in a solution of ammonia 7N in MeOH **(24.8 mL)** and stirred at 120°C under pressure 15 bar for 5h. After that time, the crude was cooled, evaporated, neutralized by addition of 100 mL of K_2CO_3 10% solution and stir for 15 minutes. Then, it was extracted with CH_2Cl_2, dried over Na_2SO_4, filtered and reduced under pressure conditions to afford **1783 mg** of **10 (96%).**

^{1}H NMR (400 MHz, $CDCl_3$) δ 7.30 (d, J = 4.8 Hz, 4H), 7.23 (s, 1H), 3.47 (d, J = 1.7 Hz, 2H), 2.86 (d, J = 11.3 Hz, 2H), 2.62 (d, J = 47.7 Hz, 1H), 1.91 (t, J = 10.8 Hz, 2H), 1.64 (d, J = 9.3 Hz, 2H), 1.51 – 1.37 (m, 2H), 1.30 – 1.18 (m, 6H).

^{13}C NMR (101 MHz, $CDCl_3$) δ 138.74, 129.34, 128.21, 126.95, 63.68, 54.08, 35.83, 34.46, 33.98, 32.57, 27.56.

4-(1-benzylpiperidin-4-yl)butan-1-amine (11).

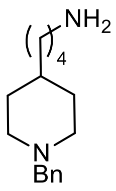

11 $C_{16}H_{26}N_2$

MW: 246.209 g/mol

11 was synthesized starting from *1-benzyl-4-(3-chloropropyl)piperidine* **11f** which, in turn, was prepared in five steps as described by Choi et al[1] and Bolea et al [2]. **11f (1 Eq., 4.41 mmol, 1086 mg)** was dissolved in a solution of ammonia 7N in MeOH **(10mL)** and stirred at 120°C under pressure 15 bar for 5h. After that time, the crude was cooled, evaporated, neutralized by addition of 100 mL of K_2CO_3 10% solution and stir for 15 minutes. Then, it was extracted with CH_2Cl_2, dried over Na_2SO_4, filtered and reduced under pressure conditions to afford **1031 mg** of **11 (95%).**

^{1}H NMR (400 MHz, $CDCl_3$) δ 7.30 (d, J = 4.3 Hz, 4H), 7.26 – 7.20 (m, 1H), 3.47 (d, J = 2.0 Hz, 1H), 2.86 (d, J = 10.5 Hz, 2H), 2.67 (t, J = 6.9 Hz, 1H), 2.58 (t, J = 6.9 Hz, 1H), 1.96

– 1.86 (m, 2H), 1.67 – 1.57 (m, J = 11.4 Hz, 3H), 1.51 – 1.38 (m, 2H), 1.29 (d, J = 12.1 Hz, 2H), 1.26 – 1.15 (m, 4H).

^{13}C NMR (101 MHz, CDCl$_3$) δ 138.72, 129.39, 128.25, 127.00, 63.71, 54.13, 50.09, 42.39, 36.63, 36.61, 35.87, 35.81, 32.54, 32.53, 24.71, 24.23.

General Procedure A (Synthesis of isocyanides 4-7):

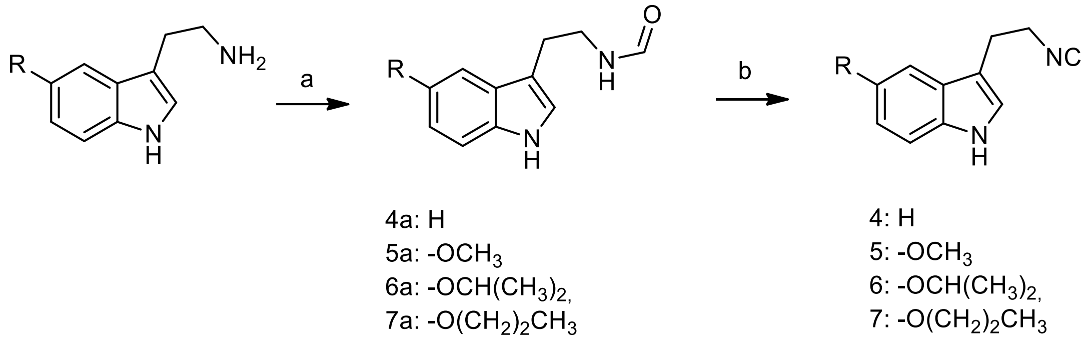

Scheme for isocyanides (4-7) synthesis. ***Reagents and conditions:*** (a) Ethyl formate, reflux, 4h; (b) Burgess reagent, dry CHCl$_2$, 1h.

General procedure A.1 – Synthesis of N-formamides derivatives (4a-7a).

Tryptamine derivative **(1 Eq.)** was dissolved in ethyl formate **(4 Eq.).** The mixture was refluxed at 70°C for 4h**.** The crude was then poured into 2N HCl solution and extracted three times with CH_2Cl_2. Organic layers were joined, washed with NaOH 5% solution, dried over Na_2SO_4, filtered, and concentrated under pressure conditions, to afford corresponding formamide with yields from **91** to **99%.**

General procedure A.2 – Synthesis of isocyanides derivatives (4-7).

Commercially available burgess reagent **(1.5 Eq.)** was added to a solution of corresponding *N*-formamide derivative, synthesized from ***general procedure A.1*** **(1 Eq.),** in freshly distilled CH_2Cl_2 (1 mL/mmol) under argon. The resulting mixture was heated to reflux at 60°C for 1h preserving anhydride conditions. After that, the crude was poured into 20 mL of CH_2Cl_2, washed twice with water, dried over Na_2SO_4, filtered and concentrated under pressure conditions to be purified by flash column chromatography at 100% CH_2Cl_2, affording yields from **51 to 59%.**

***N*-(2-(1H-indol-3-yl)ethyl)formamide (4a).**

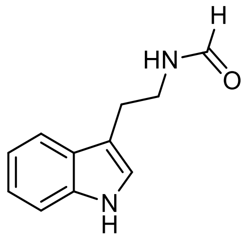

4a $C_{11}H_{12}N_2O$

MW: 188.095 g/mol

The crude was prepared according to ***general procedure A.1***, starting from commercially available tryptamine **(1 Eq., 12.48 mmol, 2000 mg)** that was suspended in ethyl formate **(3.97 Eq., 49.51 mmol, 4 mL)** to afford **2228.93 mg** of **4a** as a dark brown oil **(95%).** The crude was used for the synthesis of **4** without further purification.

***N*-(2-(5-methoxy-1H-indol-3-yl)ethyl)formamide (5a).**

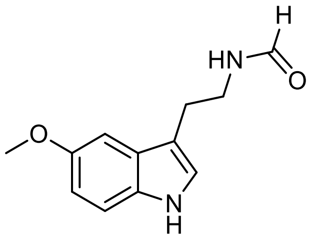

5a $C_{12}H_{14}N_2O_2$

MW: 218.016 g/mol

The crude was prepared according to ***general procedure A.1***, starting from commercially available 5-methoxytryptamine **(1 Eq., 10.51 mmol, 2000 mg)** that was suspended in ethyl formate **(4.7 Eq., 49.51 mmol, 4 mL)** to afford **2130.03 mg** of **5a** as a light brown powder **(93%).** The crude was used for the synthesis of **5** without further purification.

***N*-(2-(5-isopropoxy-1H-indol-3-yl)ethyl)formamide (6a).**

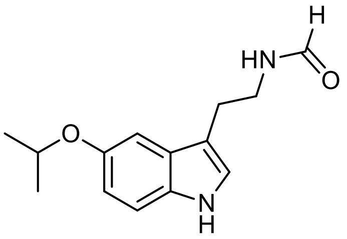

6a $C_{14}H_{18}N_2O_2$

MW: 246.105 g/mol

6a was prepared according to ***general procedure A.1*** starting from 5-*iso*propyloxytryptamine, which in turn was synthsized as described by Choi et *al* [1]and benchekroun et *al* [3] . 5-*iso*propyloxytryptamine **(1 Eq., 6.03 mmol, 1400 mg)** was suspended in ethyl formate **(4.7 Eq., 49.51 mmol, 4 mL)** to afford **1452.50 mg** of **6a** as dark brown oil **(92%).** The crude was used for the synthesis of **6** without further purification.

***N*-(2-(5-propoxy-1H-indol-3-yl)ethyl)formamide (7a).**

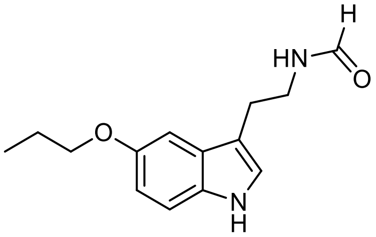

7a $C_{14}H_{18}N_2O_2$

MW: 246.105 g/mol

7a was prepared accroding to ***general procedure A.1***, starting from 5-propyloxytryptamine, which in turn was synthesized as described in by Choi et *al* [1]and benchekroun et *al* [3]. 5-propyloxytryptamine **(1 Eq., 2.99 mmol, 652 mg)** was suspended in ethyl formate **(4.7 Eq., 49.51 mmol, 4 mL)** to afford **675 mg** of **7a** as a light brown powder **(91%).** The crude was used for the synthesis of **7** without further purification.

3-(2-isocyanoethyl)-1H-indole (4)

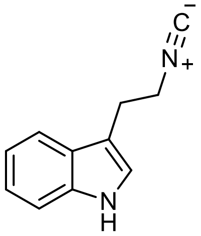

4 $C_{11}H_{10}N_2$

MW: 170.095 g/mol

The crude was prepared according to ***general procedure A.2***. Starting from **4a (1 Eq., 11.85 mmol, 2228.93 mg)** and burgess reagent **(1.5 Eq., 17.78 mmol, 4837.93 mg), 4** was afforded as a light brown powder **(1248.20 mg, 56%).**

^{1}H NMR (400 MHz, $CDCl_3$) δ 8.09 (s, 1H), 7.56 (d, J = 7.8 Hz, 1H), 7.40 (d, J = 8.1 Hz, 1H), 7.26 – 7.19 (m, 1H), 7.19 – 7.10 (m, 2H), 3.72 – 3.63 (m, 2H), 3.23 – 3.12 (m, 2H).

^{13}C NMR (101 MHz, $CDCl_3$) δ 156.15, 136.37, 126.88, 122.71, 122.53, 119.87, 118.32, 111.54, 111.22, 42.47, 25.97.

3-(2-isocyanoethyl)-5-methoxy-1H-indole (5)

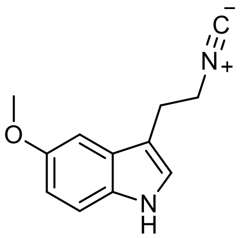

5 $C_{12}H_{12}N_2O$

MW: 200.091 g/mol

The crude was prepared according to ***general procedure A.2***. Starting from **5a (1Eq., 9.77 mmol, 2130.03 mg)** and burgess reagent (**1.5 Eq., 15.65 mmol, 3491.60 mg), 5** was afforded as a light brown powder. **(1153.16 mg, 59%)**

^{1}H NMR (400 MHz, $CDCl_3$) δ 7.98 (s, 1H), 7.28 (d, J = 8.8 Hz, 1H), 7.12 (d, J = 2.4 Hz, 1H), 6.98 (d, J = 2.4 Hz, 1H), 6.89 (dd, J = 8.8, 2.4 Hz, 1H), 3.87 (s, 3H), 3.69 – 3.62 (m, 2H), 3.14 (dd, J = 9.7, 4.4 Hz, 2H).

^{13}C NMR (101 MHz, $CDCl_3$) δ 156.2, 154.43, 131.57, 127.39, 123.56, 112.69, 112.37, 110.97, 100.37, 77.58, 77.26, 76.94, 56.19, 42.47, 26.06.

3-(2-isocyanoethyl)-5-*iso*propoxy-1H-indole (6)

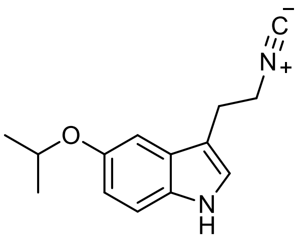

6 $C_{14}H_{16}N_2O$

MW: 228.130 g/mol

The crude was prepared according to ***general procedure A.2***. Starting from **6a (1 Eq., 5.90 mmol, 1452.50 mg)** and burgess reagent (**1.5 Eq., 8.85 mmol, 2109.61 mg), 6** was afforded as a dark brown oil **(794.37, 59%).**

^{1}H NMR (400 MHz, $CDCl_3$) δ 8.03 (s, 1H), 7.26 (d, J = 8.8 Hz, 1H), 7.10 (d, J = 2.3 Hz, 1H), 7.03 (d, J = 2.2 Hz, 1H), 6.88 (dd, J = 8.8, 2.3 Hz, 1H), 4.55 (dt, J = 12.1, 6.1 Hz, 1H), 3.65 (dd, J = 9.8, 4.3 Hz, 2H), 3.12 (t, J = 7.0 Hz, 2H), 1.37 (d, J = 6.1 Hz, 6H).

^{13}C NMR (101 MHz, $CDCl_3$) δ 156.03, 152.17, 131.77, 127.42, 123.48, 114.55, 112.12, 110.75, 104.47, 71.59, 42.37, 25.97, 22.37.

3-(2-isocyanoethyl)-5-propoxy-1H-indole (7)

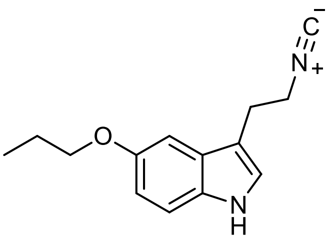

7 $C_{14}H_{16}N_2O$

MW: 228.130 g/mol

The crude was prepared according to ***general procedure A.2***. Starting from 7**a (1 Eq., 2.74 mmol, 675 mg)** and burgess reagent (**1.5 Eq., 4.11 mmol, 980.37 mg), 7** was afforded as a light brown powder **(324.02 mg, 51%).**

^{1}H NMR (400 MHz, DMSO) δ 10.73 (s, 1H), 7.23 (d, J = 8.7 Hz, 1H), 7.19 (d, J = 2.4 Hz, 1H), 7.07 (d, J = 2.3 Hz, 1H), 6.72 (dd, J = 8.7, 2.4 Hz, 1H), 3.92 (t, J = 6.6 Hz, 2H), 3.78 – 3.68 (m, 2H), 3.00 (ddd, J = 6.8, 4.9, 2.0 Hz, 2H), 1.79 – 1.68 (m, 2H), 1.00 (t, J = 7.4 Hz, 3H).

^{13}C NMR (101 MHz, DMSO) δ 155.38, 152.44, 131.29, 127.13, 124.13, 112.02, 111.73, 109.59, 101.18, 69.45, 42.05, 25.17, 22.31, 10.58.

General Procedure B (Ugi Adducts 14a-14p):

Paraformaldehyde (1 Eq.) was added to a solution of benzylamine (1 Eq.) in CH_2Cl_2:MeOH (1:3 v/v, 0.8 mmol/mL). The resulting mixture was stirred for 1h at room temperature. Then, 2-chromone-carboxylic acid (1 Eq.) and finally corresponding isocyanide (1 Eq.) were added. The reaction mixture was stirred for 24h at 60°C. The crude product was purified by flash column chromatography to afford the corresponding Ugi adduct.

***N*-(2-((2-(1H-indol-3-yl)ethyl)amino)-2-oxoethyl)-*N*-((1-benzylpiperidin-4-yl)methyl)-4-oxo-4H-chromene-2-carboxamide (14a).**

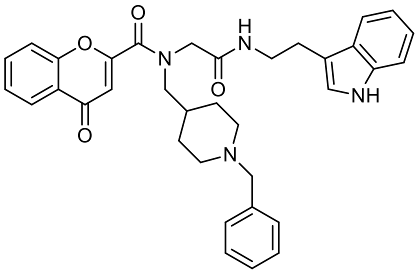

14a $C_{35}H_{36}N_4O_4$

MW: 576.274 g/mol

The crude was prepared according to ***general procedure B*** starting from commercial *1-benzylpiperidin-4-yl)methanamine* **8** (204.16 mg, 1 mmol), paraformaldehyde **13** (30.03 mg, 1

mmol), 2-chromone-carboxylic acid **12** (190.15 mg, 1 mmol) and *3-(2-isocyanoethyl)-1H-indole* **4** (170.00 mg, 1 mmol) in 10mL of CH_2Cl_2:MeOH (1:3 v/v, 0.8 mmol/mL). After 24h, the crude was purified by flash column at 9:1 CH_2Cl_2:MeOH, to afford **14a** as a light orange foam **(125 mg, 21.69%).**

^{1}H NMR (400 MHz, $CDCl_3$) δ 8.38 (s, 1H), 8.18 (d, J = 7.5 Hz, 1H), 7.72 (t, J = 7.6 Hz, 1H), 7.58 (d, J = 7.6 Hz, 1H), 7.47 (dd, J = 13.3, 5.6 Hz, 1H), 7.41 – 7.34 (m, 1H), 7.30 (dd, J = 14.8, 9.3 Hz, 4H), 7.25 – 7.19 (m, 2H), 7.14 (t, J = 7.5 Hz, 1H), 7.09 – 7.01 (m, 2H), 6.41 (s, 1H), 6.13 (d, J = 6.3 Hz, 1H), 3.99 (s, 2H), 3.65 (dd, J = 11.7, 5.8 Hz, 2H), 3.45 (s, 2H), 3.19 (d, J = 7.3 Hz, 2H), 3.01 (t, J = 6.5 Hz, 2H), 2.81 (d, J = 9.5 Hz, 2H), 1.87 (t, J = 10.7 Hz, 2H), 1.74 (s, 1H), 1.68 – 1.59 (m, 1H), 1.49 (d, J = 1.4 Hz, 2H), 1.01 (dd, J = 21.6, 11.3 Hz, 1H).

^{13}C NMR (101 MHz, $CDCl_3$) δ 177.46, 167.79, 163.72, 157.59, 155.74, 138.27, 136.65, 135.92, 134.73, 129.30, 128.34, 127.28, 127.18, 126.26, 126.04, 124.38, 122.49, 119.75, 118.76, 118.34, 112.59, 112.23, 111.64, 63.34, 56.29, 53.08, 51.03, 39.65, 34.70, 29.94, 25.05.

HRMS ESI-TOF [M+H]+ m/z calcd. for $C_{35}H_{37}N_4O_4$: 577.2783, found: 577.2809.

***N*-((1-benzylpiperidin-4-yl)methyl)-*N*-(2-((2-(5-ethyl-1H-indol-3-yl)ethyl)amino)-2-oxoethyl)-4-oxo-4H-chromene-2-carboxamide (14b).**

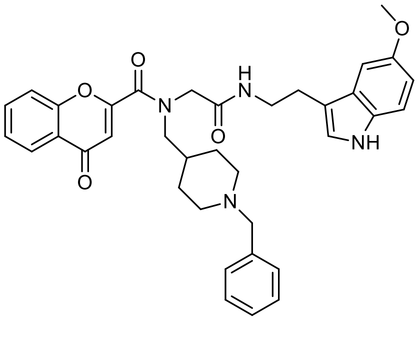

14b $C_{36}H_{38}N_4O_5$

MW: 606.284 g/mol

The crude was prepared according to ***general procedure B*** starting from commercial *1-benzylpiperidin-4-yl)methanamine* **8** (204.16 mg, 1 mmol), paraformaldehyde **13** (30.03 mg, 1 mmol), 2-chromone-carboxylic acid **12** (190.15 mg, 1 mmol) and *3-(2-isocyanoethyl)-5-methoxy-1H-indole* **5** (200.00 mg, 1 mmol) in 10mL of CH_2Cl_2:MeOH (1:3 v/v, 0.8 mmol/mL).

After 24h, the crude was purified by flash column at 9:1 CH_2Cl_2:MeOH, to afford **14b** as a yellow foam **(41 mg, 7%).**

^{1}H NMR (400 MHz, $CDCl_3$) δ 8.23 (s, 1H), 8.18 (d, J = 7.8 Hz, 1H), 7.65 (t, J = 7.5 Hz, 1H), 7.48 (t, J = 7.5 Hz, 1H), 7.39 (d, J = 8.3 Hz, 1H), 7.29 (d, J = 9.4 Hz, 2H), 7.25 – 7.15 (m, 4H), 6.99 (d, J = 14.3 Hz, 2H), 6.81 (dd, J = 8.8, 2.1 Hz, 1H), 6.42 (s, 1H), 6.12 (t, J = 6.3 Hz, 1H), 4.01 (s, 2H), 3.84 (s, 3H), 3.64 (dd, J = 11.9, 5.9 Hz, 2H), 3.46 (s, 2H), 3.21 (d, J = 7.2 Hz, 2H), 2.98 (t, J = 6.5 Hz, 2H), 2.82 (d, J = 9.2 Hz, 2H), 1.89 (t, J = 11.0 Hz, 2H), 1.77 – 1.61 (m, 2H), 1.51 (d, J = 13.1 Hz, 2H), 1.03 (dd, J = 21.7, 11.7 Hz, 1H).

^{13}C NMR (101 MHz, $CDCl_3$) δ 177.45, 167.79, 163.73, 157.55, 155.74, 154.27, 138.46, 134.73, 131.77, 129.30, 128.35, 127.71, 127.20, 126.26, 126.05, 124.39, 123.21, 118.36, 114.21, 112.62, 100.63, 63.31, 56.31, 56.03, 53.08, 51.08, 39.52, 34.72, 29.94, 25.06.

HRMS ESI-TOF [M+H]+ m/z calcd. for $C_{36}H_{39}N_4O_5$: 607.2915, found: 607.2888.

***N*-((1-benzylpiperidin-4-yl)methyl)-*N*-(2-((2-(5-isopropoxy-1H-indol-3-yl)ethyl)amino)-2-oxoethyl)-4-oxo-4H-chromene-2-carboxamide (14c).**

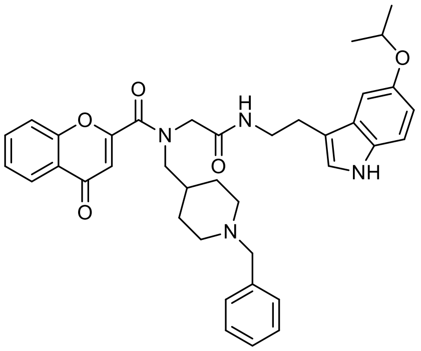

14c $C_{38}H_{42}N_4O_5$

MW: 634.316 g/mol

The crude was prepared according to ***general procedure B*** starting from commercial *1-benzylpiperidin-4-yl)methanamine* **8** (204.16 mg, 1 mmol), paraformaldehyde **13** (30.03 mg, 1 mmol), 2-chromone-carboxylic acid **12** (190.15 mg, 1 mmol) and *3-(2-isocyanoethyl)-5-isopropoxy-1H-indole* **6** (228.00 mg, 1 mmol) in 10mL of CH_2Cl_2:MeOH (1:3 v/v, 0.8 mmol/mL). After 24h, the crude was purified by flash column at 9:1 CH_2Cl_2:MeOH, to afford **14c** as a yellow foam **(104.30 mg, 16.44%).**

^{1}H NMR (400 MHz, CDCl$_3$) δ 8.28 – 8.11 (m, J = 7.8 Hz, 2H), 7.66 (t, J = 7.8 Hz, 1H), 7.48 (t, J = 7.5 Hz, 1H), 7.44 – 7.37 (m, 1H), 7.36 – 7.27 (m, 3H), 7.25 – 7.15 (m, 3H), 7.09 – 6.97 (m, 2H), 6.81 (dd, J = 8.7, 2.2 Hz, 1H), 6.39 – 6.28 (m, 2H), 4.56 – 4.46 (m, 1H), 4.00 (s, 2H), 3.63 (dd, J = 11.9, 6.0 Hz, 2H), 3.44 (s, 2H), 3.22 (d, J = 7.4 Hz, 2H), 2.96 (t, J = 6.5 Hz, 2H), 2.76 (d, J = 10.2 Hz, 2H), 1.85 (t, J = 10.6 Hz, 2H), 1.71 – 1.62 (m, 1H), 1.47 (d, J = 10.1 Hz, 2H), 1.33 (d, J = 6.1 Hz, 7H), 1.10 – 0.99 (m, J = 11.8 Hz, 1H).

^{13}C NMR (101 MHz, CDCl$_3$) δ 177.50, 167.75, 163.71, 157.59, 155.75, 152.15, 135.92, 134.76, 132.08, 129.33, 128.36, 127.82, 126.27, 126.04, 124.37, 123.25, 120.54, 118.38, 114.60, 112.24, 112.18, 104.76, 71.42, 71.27, 63.27, 56.28, 53.06, 51.05, 39.48, 34.69, 29.84, 25.09, 22.43, 22.38.

HRMS ESI-TOF [M+H]+ m/z calcd. for $C_{38}H_{43}N_4O_5$: 635.3228, found: 635.3210.

***N*-((1-benzylpiperidin-4-yl)methyl)-4-oxo-*N*-(2-oxo-2-((2-(5-propoxy-1H-indol-3-yl)ethyl)amino)ethyl)-4H-chromene-2-carboxamide (14d).**

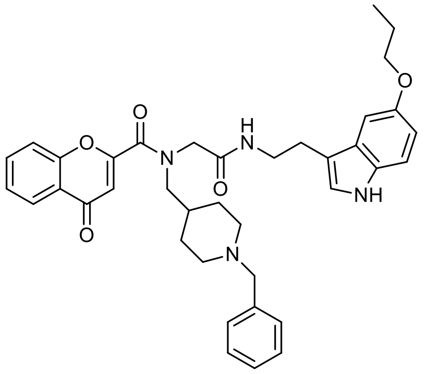

14d $C_{38}H_{42}N_4O_5$

MW: 634.316 g/mol

The crude was prepared according to ***general procedure B*** starting from commercial *1-benzylpiperidin-4-yl)methanamine* **8** (204.16 mg, 1 mmol), paraformaldehyde **13** (30.03 mg, 1 mmol), 2-chromone-carboxylic acid **12** (190.15 mg, 1 mmol) and *3-(2-isocyanoethyl)-5-propoxy-1H-indole* **7** (228.00 mg, 1 mmol) in 10mL of CH_2Cl_2:MeOH (1:3 v/v, 0.8 mmol/mL). After 24h, the crude was purified by flash column at 9:1 CH_2Cl_2:MeOH, to afford **14d** as a gold foam **(96 mg, 15.13%).**

^{1}H NMR (400 MHz, CDCl$_3$) δ 8.18 (d, J = 7.7 Hz, 1H), 8.11 (s, 1H), 7.66 (t, J = 7.6 Hz, 1H), 7.48 (t, J = 7.6 Hz, 1H), 7.39 (d, J = 8.4 Hz, 1H), 7.34 – 7.27 (m, 3H), 7.25 – 7.16 (m,

J = 14.3, 8.6 Hz, 3H), 6.99 (d, J = 16.2 Hz, 2H), 6.82 (dd, J = 8.8, 2.2 Hz, 1H), 6.42 (s, 1H), 6.10 (s, 1H), 4.06 (s, 1H), 4.00 – 3.90 (m, 3H), 3.69 – 3.60 (m, 2H), 3.41 (s, 1H), 3.20 (d, J = 7.0 Hz, 2H), 2.97 (t, J = 6.5 Hz, 2H), 2.81 (d, J = 9.1 Hz, 2H), 1.91 – 1.76 (m, 4H), 1.70 – 1.60 (m, 2H), 1.60 – 1.51 (m, 1H), 1.46 (d, J = 12.2 Hz, 2H), 1.05 (t, J = 7.4 Hz, 4H).

^{13}C NMR (101 MHz, $CDCl_3$) δ 177.48, 167.77, 163.72, 157.56, 155.75, 153.75, 134.74, 131.79, 129.30, 128.35, 127.72, 127.19, 126.27, 126.05, 124.39, 123.17, 118.36, 113.15, 112.29, 101.79, 70.53, 63.31, 56.28, 53.08, 51.05, 39.49, 34.72, 29.94, 29.85, 25.06, 22.97, 10.80.

HRMS ESI-TOF [M+H]+ m/z calcd. for $C_{38}H_{43}N_4O_5$: 635.3228, found: 635.3211.

***N*-(2-((2-(1H-indol-3-yl)ethyl)amino)-2-oxoethyl)-*N*-(2-(1-benzylpiperidin-4-yl)ethyl)-4-oxo-4H-chromene-2-carboxamide (14e).**

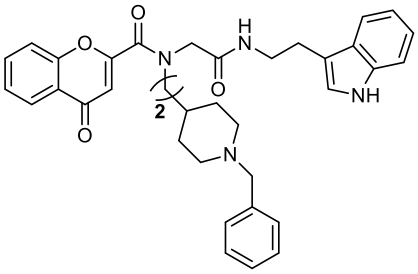

14e $C_{36}H_{38}N_4O_4$
MW: 590.289 g/mol

The crude was prepared according to ***general procedure B*** starting from commercial *4-(2-aminoethyl)-1-benzylpiperidine* **9** (436.68 mg, 2 mmol), paraformaldehyde **13** (60 mg, 2 mmol), 2-chromone-carboxylic acid **12** (380.2 mg, 2 mmol) and *3-(2-isocyanoethyl)-1H-indole* **4** (340 mg, 2 mmol) in 10mL of CH_2Cl_2:MeOH (1:3 v/v, 0.8 mmol/mL). After 24h, the crude was purified by flash column at 9:1 CH_2Cl_2:MeOH, to afford **14e** as a light yellow powder **(391 mg, 33.11%).**

^{1}H NMR (400 MHz, $CDCl_3$) δ 8.73 – 8.44 (m, 1H), 8.16 (d, J = 7.4 Hz, 1H), 7.71 (t, J = 7.6 Hz, 1H), 7.58 (d, J = 7.6 Hz, 1H), 7.45 (t, J = 7.5 Hz, 1H), 7.35 (d, J = 8.6 Hz, 1H), 7.33 – 7.26 (m, J = 13.4 Hz, 4H), 7.24 – 7.21 (m, 1H), 7.19 – 6.95 (m, 4H), 6.48 (s, 1H), 6.18 (t, J = 6.2 Hz, 1H), 3.96 (s, 2H), 3.71 – 3.62 (m, J = 17.9, 11.9 Hz, 2H), 3.42 (s, 1H), 3.30 – 3.19 (m,

2H), 3.01 (t, J = 6.3 Hz, 2H), 2.83 (d, J = 10.6 Hz, 2H), 1.99 (s, 1H), 1.82 (t, J = 10.8 Hz, 1H), 1.59 – 1.51 (m, J = 8.2 Hz, 2H), 1.42 (s, 4H), 1.26 (s, 2H).

^{13}C NMR (101 MHz, CDCl$_3$) δ 177.49, 167.68, 163.17, 157.63, 155.57, 138.32, 136.60, 134.73, 129.31, 128.30, 127.39, 127.13, 126.20, 126.03, 124.31, 122.48, 122.30, 119.60, 118.71, 118.21, 112.50, 112.10, 111.61, 63.44, 53.65, 50.22, 48.54, 39.67, 35.07, 33.60, 32.06, 25.03.

HRMS ESI-TOF [M+H]+ m/z calcd. for $C_{37}H_{39}N_4O_4$: 591.2966, found: 591.2954.

***N*-(2-(1-benzylpiperidin-4-yl)ethyl)-*N*-(2-((2-(5-methoxy-1H-indol-3-yl)ethyl)amino)-2-oxoethyl)-4-oxo-4H-chromene-2-carboxamide (14f).**

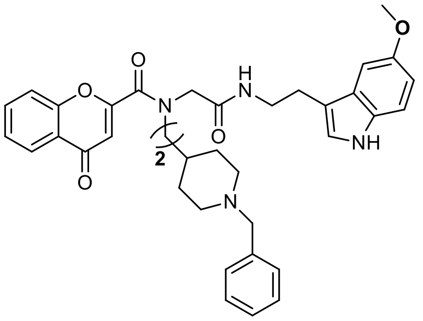

14f $C_{37}H_{40}N_4O_5$

MW: 620.737 g/mol

The crude was prepared according to ***general procedure B*** starting from commercial *4-(2-aminoethyl)-1-benzylpiperidine* **9** (436.68 mg, 2 mmol), paraformaldehyde **13** (60 mg, 2 mmol), 2-chromone-carboxylic acid **12** (380.2 mg, 2 mmol) and *3-(2-isocyanoethyl)-5-methoxy-1H-indole* **5** (400 mg, 2 mmol) in 10mL of CH_2Cl_2:MeOH (1:3 v/v, 0.8 mmol/mL). After 24h, the crude was purified by flash column at 9:1 CH_2Cl_2:MeOH, to afford **14f** as a gold foam **(92 mg, 14,82%**).

^{1}H NMR (400 MHz, CDCl$_3$) δ 8.37 (s, 1H), 8.18 (d, J = 7.6 Hz, 1H), 7.66 (t, J = 50.0, 7.2 Hz, 1H), 7.47 (t, J = 7.5 Hz, 1H), 7.37 (d, J = 8.5 Hz, 1H), 7.33 – 7.27 (m, 4H), 7.24 – 7.11 (m, 2H), 7.07 – 6.93 (m, 2H), 6.80 (dd, J = 8.8, 2.3 Hz, 1H), 6.47 (s, 1H), 6.10 (t, J = 6.2 Hz, 1H), 3.98 (s, 2H), 3.82 (s, 3H), 3.73 – 3.61 (m, 2H), 3.52 – 3.48 (m, 1H), 3.43 (s, 1H), 3.30 – 3.22 (m, 2H), 3.04 – 2.95 (m, 2H), 2.84 (d, J = 10.5 Hz, 2H), 1.95 (s, 1H), 1.88 – 1.76 (m, 3H), 1.63 – 1.52 (m, 2H), 1.43 (d, J = 11.3 Hz, 1H), 1.20 – 1.09 (m, 2H).

^{13}C NMR (101 MHz, CDCl$_3$) δ 177.48, 167.73, 163.22, 157.58, 155.61, 154.24, 138.29, 134.74, 131.75, 129.33, 128.32, 127.80, 127.15, 126.24, 126.09, 124.37, 123.22, 118.24, 112.50, 112.34, 112.27, 112.14, 100.67, 63.44, 56.03, 53.64, 50.42, 48.63, 39.49, 35.14, 33.64, 32.08, 25.04.

HRMS ESI-TOF [M+H]+ m/z calcd. for $C_{37}H_{41}N_4O_5$: 621.3071 , found: 621.3058.

***N*-(2-(1-benzylpiperidin-4-yl)ethyl)-*N*-(2-((2-(5-isopropoxy-1H-indol-3-yl)ethyl)amino)-2-oxoethyl)-4-oxo-4H-chromene-2-carboxamide (14g).**

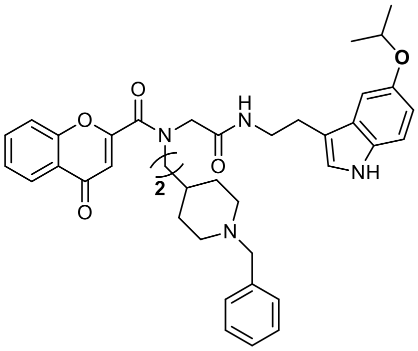

14g $C_{39}H_{44}N_4O_5$

MW: 648.331 g/mol

The crude was prepared according to ***general procedure B*** starting from commercial *4-(2-aminoethyl)-1-benzylpiperidine* **9** (436.68 mg, 2 mmol), paraformaldehyde **13** (60 mg, 2 mmol), 2-chromone-carboxylic acid **12** (380.2 mg, 2 mmol) and *3-(2-isocyanoethyl)-5-isopropoxy-1H-indole* **6** (456 mg, 2 mmol) in 10mL of CH_2Cl_2:MeOH (1:3 v/v, 0.8 mmol/mL). After 24h, the crude was purified by flash column at 9:1 CH_2Cl_2:MeOH, to afford **14g** as a light yellow foam **(227 mg, 17.51%)**.

^{1}H NMR (400 MHz, CDCl$_3$) δ 8.39 (s, 1H), 8.18 (d, J = 7.6 Hz, 1H), 7.65 (t, J = 7.0 Hz, 1H), 7.46 (t, J = 7.4 Hz, 1H), 7.39 (t, J = 10.0 Hz, 1H), 7.31 – 7.27 (m, 4H), 7.24 – 7.10 (m, 2H), 7.08 – 6.91 (m, 2H), 6.80 (dd, J = 8.8, 2.2 Hz, 1H), 6.48 (s, 1H), 6.11 (t, J = 6.2 Hz, 1H), 4.49 (m, J = 12.1, 6.1 Hz, 1H), 3.98 (s, 2H), 3.72 – 3.59 (m, 2H), 3.42 (s, 1H), 3.34 – 3.25 (m, 2H), 2.96 (t, J = 6.1 Hz, 2H), 2.83 (d, J = 10.4 Hz, 2H), 1.93 (s, 1H), 1.88 – 1.72 (m, 3H), 1.62 – 1.52 (m, 2H), 1.51 – 1.40 (m, 2H), 1.32 (d, J = 5.9 Hz, 7H), 1.17 – 1.09 (m, 1H).

^{13}C NMR (101 MHz, CDCl$_3$) δ 177.39, 167.59, 163.11, 157.52, 155.52, 152.03, 138.29, 134.64, 131.95, 129.24, 128.20, 127.77, 127.02, 126.11, 125.97, 124.26, 123.14,

118.16, 114.46, 112.07, 104.76, 71.40, 63.37, 53.46, 50.26, 48.52, 39.37, 35.06, 33.52, 32.02, 24.97, 22.31.

HRMS ESI-TOF [M+H]+ m/z calcd. for $C_{39}H_{45}N_4O_5$: 649.3384, found: 649.3365.

***N*-(2-(1-benzylpiperidin-4-yl)ethyl)-4-oxo-*N*-(2-oxo-2-((2-(5-propoxy-1H-indol-3-yl)ethyl)amino)ethyl)-4H-chromene-2-carboxamide (14h).**

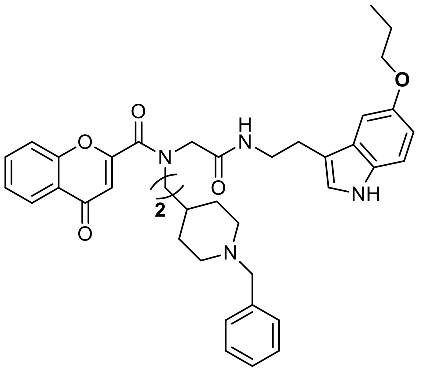

14h $C_{39}H_{44}N_4O_5$

MW: 648.331 g/mol

The crude was prepared according to ***general procedure B*** starting from commercial *4-(2-aminoethyl)-1-benzylpiperidine* **9** (436.68 mg, 2 mmol), paraformaldehyde **13** (60 mg, 2 mmol), 2-chromone-carboxylic acid **12** (380.2 mg, 2 mmol) and *3-(2-isocyanoethyl)-5-propoxy-1H-indole* **7** (456 mg, 2 mmol) in 10mL of CH_2Cl_2:MeOH (1:3 v/v, **0.8 mmol/mL**). After 24h, the crude was purified by flash column at 9:1 CH_2Cl_2:MeOH, to afford **14h** as a light yellow foam **(92.0 mg, 7.1%**).

^{1}H NMR (400 MHz, $CDCl_3$) δ 8.37 (s, 1H), 8.18 (d, J = 7.1 Hz, 1H), 7.69 (d, J = 7.3 Hz, 1H), 7.46 (t, J = 7.4 Hz, 1H), 7.37 (d, J = 8.4 Hz, 1H), 7.34 – 7.27 (m, 3H), 7.23 – 7.11 (m, 2H), 6.99 (d, J = 15.1 Hz, 2H), 6.80 (d, J = 8.6 Hz, 1H), 6.47 (s, 1H), 6.10 (t, J = 6.2 Hz, 1H), 4.03 (s, 1H), 3.93 (t, J = 6.5 Hz, 3H), 3.76 – 3.61 (m, 2H), 3.42 (s, 1H), 3.31 – 3.21 (m, 2H), 2.98 (d, J = 6.1 Hz, 2H), 2.83 (d, J = 10.2 Hz, 2H), 1.94 (s, 1H), 1.80 (q, J = 17.5, 10.5 Hz, 4H), 1.75 (d, J = 5.0 Hz, 1H), 1.63 – 1.51 (m, J = 16.3 Hz, 2H), 1.51 – 1.39 (m, 2H), 1.29 – 1.24 (m, 1H), 1.13 (d, J = 8.9 Hz, 1H), 1.04 (t, J = 7.4 Hz, 3H).

^{13}C NMR (101 MHz, $CDCl_3$) δ 177.40, 167.60, 163.11, 157.50, 155.51, 153.61, 138.24, 134.64, 131.66, 129.22, 128.21, 127.69, 127.04, 126.13, 125.97, 124.26, 123.08,

118.15, 112.96, 112.15, 112.09, 112.02, 101.72, 70.46, 63.36, 53.46, 50.27, 48.51, 39.33, 35.05, 33.51, 31.99, 24.93, 22.85, 10.68.

HRMS ESI-TOF [M+H]+ m/z calcd. for $C_{39}H_{45}N_4O_5$: 649.3384, found: 649.3369.

***N*-(2-((2-(1H-indol-3-yl)ethyl)amino)-2-oxoethyl)-*N*-(3-(1-benzylpiperidin-4-yl)propyl)-4-oxo-4H-chromene-2-carboxamide (14i).**

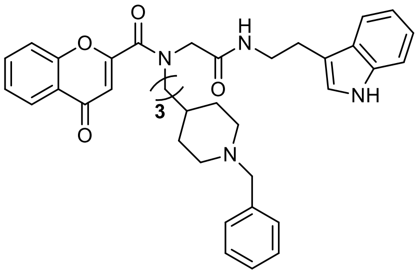

14i $C_{37}H_{40}N_4O_4$

MW: 604.305 g/mol

The crude was prepared according to ***general procedure B*** starting from *4-(1-benzylpiperidin-4-yl)propan-1-amine* **10** (232.16 mg, 1 mmol), paraformaldehyde **13** (300. mg, 1 mmol), 2-chromone-carboxylic acid **12** (190.15 mg, 1 mmol) and *3-(2-isocyanoethyl)-1H-indole* **4** (170.24 mg, 1 mmol) in 10mL of CH_2Cl_2:MeOH (1:3 v/v, 0.8 mmol/mL). After 24h, the crude was purified by flash column at 9:1 CH_2Cl_2:MeOH, to afford **14i** as a yellow foam **(57 mg, 9.43%).**

^{1}H NMR (400 MHz, $CDCl_3$) δ 8.51 (s, 1H), 8.19 (d, J = 7.9 Hz, 1H), 7.69 (t, J = 7.7 Hz, 1H), 7.56 (d, J = 8.2 Hz, 1H), 7.46 (t, J = 7.4 Hz, 1H), 7.35 – 7.21 (m, 7H), 7.14 (t, J = 7.5 Hz, 1H), 7.11 – 7.04 (m, 2H), 6.48 (s, 1H), 6.09 (s, 1H), 3.99 (s, 2H), 3.67 (dd, J = 12.0, 6.1 Hz, 2H), 3.47 (s, 2H), 3.24 (t, J = 3.2 Hz, 2H), 3.02 (t, J = 6.4 Hz, 2H), 2.84 (d, J = 11.1 Hz, 2H), 1.89 (t, J = 7.7 Hz, 2H), 1.71 – 1.55 (m, 3H), 1.51 (d, J = 10.7 Hz, 2H), 1.24 – 1.13 (m, 3H), 1.12 – 1.02 (m, J = 12.6 Hz, 1H).

^{13}C NMR (101 MHz, $CDCl_3$) δ 177.51, 167.81, 163.24, 157.66, 155.59, 138.30, 136.61, 134.75, 129.47, 128.33, 127.42, 127.17, 126.20, 126.07, 124.39, 122.38, 119.65, 118.75, 118.17, 112.57, 112.16, 111.59, 63.62, 53.81, 51.04, 50.63, 39.60, 35.32, 33.25, 32.04, 25.70, 25.06.

HRMS ESI-TOF [M+H]+ m/z calcd. for $C_{37}H_{41}N_4O_4$: 605.3122, found: 605.3105.

***N*-(3-(1-benzylpiperidin-4-yl)propyl)-*N*-(2-((2-(5-methoxy-1H-indol-3-yl)ethyl)amino)-2-oxoethyl)-4-oxo-4H-chromene-2-carboxamide (14j).**

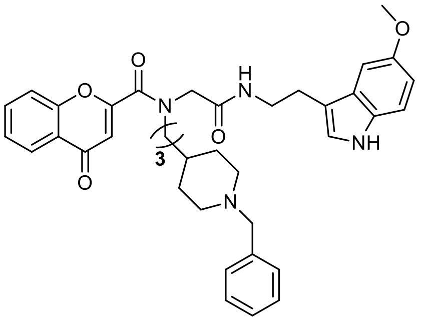

14j $C_{38}H_{42}N_4O_5$

MW: 634.315 g/mol

The crude was prepared according to ***general procedure B*** starting from *4-(1-benzylpiperidin-4-yl)propan-1-amine* **10** (232.16 mg, 1 mmol), paraformaldehyde **13** (30.03 mg, 1 mmol), 2-chromone-carboxylic acid **12** (190.15 mg, 1 mmol) and *3-(2-isocyanoethyl)-5-methoxy-1H-indole* **5** (200.04 mg, 1 mmol) in 10mL of CH_2Cl_2:MeOH (1:3 v/v, 0.8 mmol/mL). After 24h, the crude was purified by flash column at 9:1 CH_2Cl_2:MeOH, to afford **14j** as a yellow foam (**14 mg, 2.21%**).

^{1}H NMR (400 MHz, $CDCl_3$) δ 8.40 (s, 1H), 8.19 (d, J = 7.7 Hz, 1H), 7.64 (t, J = 7.5 Hz, 1H), 7.46 (t, J = 7.4 Hz, 1H), 7.34 – 7.21 (m, 6H), 7.18 (d, J = 8.9 Hz, 1H), 7.03 (d, J = 22.5 Hz, 2H), 6.80 (dd, J = 8.8, 2.2 Hz, 1H), 6.48 (s, 1H), 6.11 (s, 1H), 4.00 (s, 2H), 3.82 (s, 3H), 3.66 (dd, J = 12.0, 6.0 Hz, 2H), 3.46 (s, 2H), 3.26 (d, J = 3.2 Hz, 2H), 2.98 (t, J = 6.3 Hz, 2H), 2.84 (d, J = 11.1 Hz, 2H), 1.86 (t, J = 14.3 Hz, 2H), 1.64 – 1.47 (m, 5H), 1.19 – 1.13 (m, 2H), 1.12 – 1.03 (m, J = 6.7 Hz, 2H).

^{13}C NMR (101 MHz, $CDCl_3$) δ 177.49, 167.83, 163.25, 157.62, 155.60, 154.23, 138.40, 134.74, 131.73, 129.45, 129.36, 128.33, 127.83, 127.14, 126.21, 126.07, 124.40, 123.11, 118.17, 112.52, 112.31, 112.25, 112.20, 100.59, 63.66, 56.01, 53.83, 51.12, 50.72, 39.48, 35.34, 33.25, 32.07, 25.71, 25.05.

HRMS ESI-TOF [M+H]+ m/z calcd. for $C_{38}H_{43}N_4O_5$: 635.3228, found: 635.3210.

***N*-(3-(1-benzylpiperidin-4-yl)propyl)-*N*-(2-((2-(5-isopropoxy-1H-indol-3-yl)ethyl)amino)-2-oxoethyl)-4-oxo-4H-chromene-2-carboxamide (14k).**

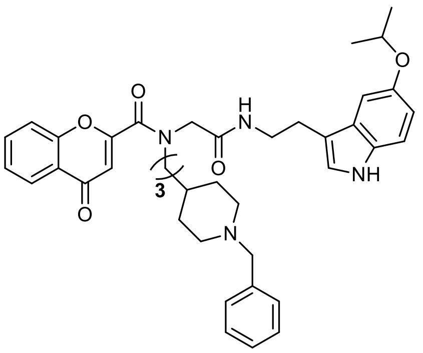

14k $C_{40}H_{46}N_4O_5$

MW: 662.331 g/mol

The crude was prepared according to ***general procedure B*** starting from *4-(1-benzylpiperidin-4-yl)propan-1-amine* **10** (173.00 mg, 1 mmol), paraformaldehyde **13** (22.15 mg, 0.75 mmol), 2-chromone-carboxylic acid **12** (142.6 mg, 0.75 mmol) and *3-(2-isocyanoethyl)-5-isopropoxy-1H-indole* **6** (170.00 mg, 0.75 mmol) in 10mL of CH_2Cl_2:MeOH (1:3 v/v, 0.8 mmol/mL). After 24h, the crude was purified by flash column at 9:1 CH_2Cl_2:MeOH, to afford **14k** as a orange foam **(14 mg, 2.82%**).

^{1}H NMR (400 MHz, $CDCl_3$) δ 8.35 (s, 1H), 8.21 (d, J = 7.5 Hz, 1H), 7.65 (t, J = 7.5 Hz, 1H), 7.49 – 7.41 (m, 1H), 7.35 – 7.28 (m, 6H), 7.19 (d, J = 8.7 Hz, 1H), 7.08 – 6.92 (m, 2H), 6.77 (d, J = 9.0 Hz, 1H), 6.49 (s, 1H), 6.07 (t, 1H), 4.53 – 4.45 (m, 1H), 4.01 (s, 2H), 3.69 – 3.59 (m, 2H), 3.51 – 3.44 (m, J = 5.9 Hz, 2H), 3.34 – 3.20 (m, 2H), 3.00 – 2.94 (m, 2H), 2.85 (d, J = 8.6 Hz, 2H), 1.99 – 1.83 (m, J = 36.6 Hz, 2H), 1.52 (d, J = 10.8 Hz, 4H), 1.31 (d, J = 6.0 Hz, 7H), 1.14 – 1.03 (m, 3H).

^{13}C NMR (101 MHz, $CDCl_3$) δ 177.39, 167.42, 163.26, 157.64, 155.32, 152.11, 138.46, 134.77, 132.04, 129.53, 128.39, 127.92, 127.28, 126.21, 126.06, 125.24, 123.14, 118.19, 118.03, 113.35, 112.17, 104.79, 71.45, 63.52, 53.77, 51.06, 50.65, 39.47, 35.32, 33.47, 32.16, 29.85, 25.09, 24.92, 22.41.

HRMS ESI-TOF [M+H]+ m/z calcd. for $C_{40}H_{47}N_4O_5$: 663.3541, found: 663.3522.

***N*-(3-(1-benzylpiperidin-4-yl)propyl)-4-oxo-*N*-(2-oxo-2-((2-(5-propoxy-1H-indol-3-yl)ethyl)amino)ethyl)-4H-chromene-2-carboxamide (14l).**

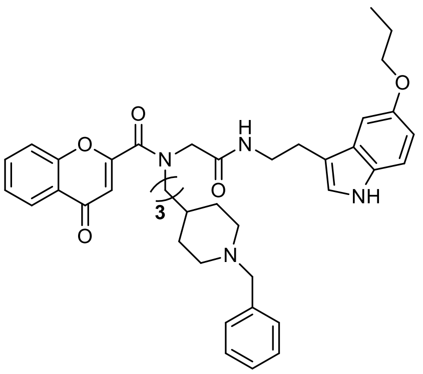

14l $C_{40}H_{46}N_4O_5$

MW: 662.331 g/mol

The crude was prepared according to ***general procedure B*** starting from *4-(1-benzylpiperidin-4-yl)propan-1-amine* **10** (232.16 mg, 1 mmol), paraformaldehyde **13** (30.03 mg, 1 mmol), 2-chromone-carboxylic acid **12** (190.15 mg, 1 mmol) and *3-(2-isocyanoethyl)-5-propoxy-1H-indole* **7** (228.00 mg, 1 mmol) in 10mL of CH_2Cl_2:MeOH (1:3 v/v, 0.8 mmol/mL). After 24h, the crude was purified by flash column at 9:1 CH_2Cl_2:MeOH, to afford **14j** as a light brown foam **(23 mg, 3.47%**).

^{1}H NMR (400 MHz, $CDCl_3$) δ 8.41 (s, 1H), 8.18 (d, J = 7.7 Hz, 1H), 7.64 (t, J = 8.1 Hz, 1H), 7.45 (t, J = 7.5 Hz, 1H), 7.35 – 7.21 (m, 6H), 7.17 (d, J = 8.7 Hz, 1H), 6.99 (d, J = 17.0 Hz, 2H), 6.80 (dd, J = 8.6, 1.7 Hz, 1H), 6.48 (s, 1H), 6.13 (s, 1H), 4.00 (s, 2H), 3.91 (t, J = 6.6 Hz, 2H), 3.64 (dd, J = 13.2, 7.5 Hz, 2H), 3.52 – 3.44 (m, 2H), 3.24 (dt, J = 16.4, 9.3 Hz, 2H), 2.97 (t, J = 6.0 Hz, 2H), 2.84 (d, J = 11.2 Hz, 2H), 1.98 – 1.74 (m, 5H), 1.66 – 1.47 (m, 4H), 1.22 – 1.13 (m, 3H), 1.11 – 0.98 (m, 4H).

^{13}C NMR (101 MHz, $CDCl_3$) δ 177.50, 167.79, 163.22, 157.62, 155.59, 153.68, 138.35, 134.74, 131.75, 129.45, 128.33, 127.84, 127.15, 126.19, 126.04, 124.39, 123.09, 118.17, 113.01, 112.22, 112.16, 101.75, 70.52, 63.63, 53.82, 51.07, 50.65, 39.48, 35.35, 33.25, 32.05, 25.73, 25.03, 22.95, 10.77.

HRMS ESI-TOF [M+H]+ m/z calcd. for $C_{40}H_{47}N_4O_5$: 663.3541, found: 663.3517.

***N*-(2-((2-(1H-indol-3-yl)ethyl)amino)-2-oxoethyl)-*N*-(4-(1-benzylpiperidin-4-yl)butyl)-4-oxo-4H-chromene-2-carboxamide (14m).**

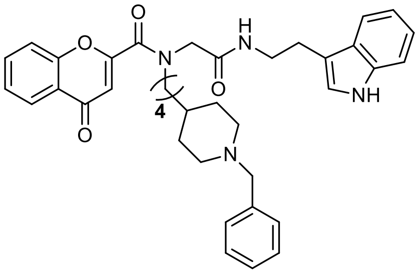

14m $C_{38}H_{42}N_4O_4$

MW: 618.321 g/mol

The crude was prepared according to ***general procedure B*** starting from *4-(1-benzylpiperidin-4-yl)butan-1-amine* **11** (246.5 mg, 1 mmol), paraformaldehyde **13** (30.03 mg, 1 mmol), 2-chromone-carboxylic acid **12** (190.15 mg, 1 mmol) and *3-(2-isocyanoethyl)-1H-indole* **4** (170.00 mg, 1 mmol) in 10mL of CH_2Cl_2:MeOH (1:3 v/v, 0.8 mmol/mL). After 24h, the crude was purified by flash column at 9:1 CH_2Cl_2:MeOH with NH_3 1%, to afford **14m** as an orange foam **(71.4 mg, 11.54%**).

^{1}H NMR (400 MHz, CDCl3) δ 8.45 (s, 1H), 8.19 (d, J = 7.6 Hz, 1H), 7.70 (dd, J = 16.8, 8.1 Hz, 1H), 7.46 (d, J = 7.1 Hz, 1H), 7.33 (dd, J = 12.2, 6.2 Hz, 7H), 7.21 – 7.11 (m, 2H), 7.10 – 6.96 (m, 2H), 6.49 (s, 1H), 6.13 (s, 1H), 3.95 (s, 2H), 3.72 – 3.60 (m, J = 18.2, 11.5, 6.6 Hz, 2H), 3.52 (t, J = 16.0 Hz, 2H), 3.32 – 3.16 (m, 2H), 3.02 (t, J = 6.3 Hz, 2H), 2.94 – 2.82 (m, 2H), 2.01 – 1.87 (m, 3H), 1.69 – 1.59 (m, 2H), 1.52 (d, J = 12.3 Hz, 3H), 1.12 (s, 5H).

^{13}C NMR (101 MHz, CDCl3) δ 177.70, 167.79, 163.23, 156.33, 155.89, 154.11, 146.88, 136.63, 134.74, 129.88, 129.61, 128.39, 128.15, 127.33, 126.24, 126.08, 124.39, 122.47, 122.37, 119.67, 118.75, 118.21, 112.47, 112.16, 111.64, 63.39, 53.78, 50.57, 39.68, 36.05, 35.49, 31.97, 29.84, 28.80, 25.07, 23.81.

HRMS ESI-TOF [M+H]+ m/z calcd. for $C_{38}H_{43}N_4O_5$: 619.3279, found: 619.3251.

***N*-(4-(1-benzylpiperidin-4-yl)butyl)-*N*-(2-((2-(5-methoxy-1H-indol-3-yl)ethyl)amino)-2-oxoethyl)-4-oxo-4H-chromene-2-carboxamide (14n).**

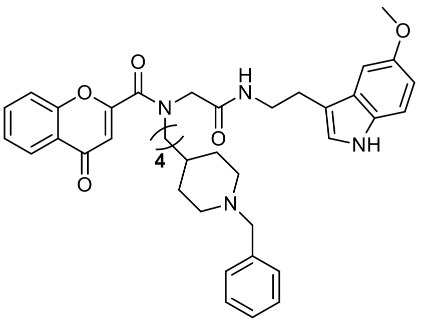

14n $C_{39}H_{44}N_4O_5$

MW: 648.337 g/mol

The crude was prepared according to ***general procedure B*** starting from *4-(1-benzylpiperidin-4-yl)butan-1-amine* **11** (203 mg, 0.82 mmol), paraformaldehyde **13** (24.73 mg, 0.82 mmol), 2-chromone-carboxylic acid **12** (166.81 mg, 0.82 mmol) and *3-(2-isocyanoethyl)-5-methoxy-1H-indole* **5** (164.50 mg, 0.82 mmol) in 10mL of CH_2Cl_2:MeOH (1:3 v/v, 0.8 mmol/mL). After 24h, the crude was purified by flash column at 9:1 CH_2Cl_2:MeOH wtih NH_3 1%, to afford **14n** as a light brown foam (**49 mg, 7.55%**).

^{1}H NMR (400 MHz, $CDCl_3$) δ 8.20 (s, 2H), 7.65 (t, J = 6.2 Hz, 1H), 7.47 (t, J = 7.5 Hz, 1H), 7.38 – 7.28 (m, 5H), 7.24 – 7.13 (m, 2H), 7.03 (d, J = 7.1 Hz, 2H), 6.80 (dd, J = 8.8, 2.4 Hz, 1H), 6.48 (s, 1H), 6.12 (s, 1H), 4.00 (s, 2H), 3.83 (s, 3H), 3.71 – 3.63 (m, 2H), 3.54 (s, 2H), 3.35 – 3.21 (m, 2H), 3.05 – 2.94 (m, J = 13.9, 7.1 Hz, 2H), 2.93 – 2.77 (m, 2H), 2.03 – 1.84 (m, 3H), 1.60 – 1.44 (m, J = 12.5 Hz, 5H), 1.13 (s, 5H).

^{13}C NMR (101 MHz, $CDCl_3$) δ 177.26, 167.81, 163.25, 154.51, 154.24, 152.74, 146.47, 136.72, 134.73, 131.77, 129.51, 128.35, 127.88, 127.23, 126.23, 126.10, 124.84, 123.21, 119.19, 118.21, 112.54, 112.35, 112.18, 100.64, 63.50, 56.04, 53.85, 50.62, 39.51, 36.14, 35.60, 32.29, 32.10, 28.88, 25.07, 23.83.

HRMS ESI-TOF [M+H]+ m/z calcd. for $C_{39}H_{45}N_4O_5$: 649.3384, found: 649.3372.

***N*-(4-(1-benzylpiperidin-4-yl)butyl)-*N*-(2-((2-(5-isopropoxy-1H-indol-3-yl)ethyl)amino)-2-oxoethyl)-4-oxo-4H-chromene-2-carboxamide (14o).**

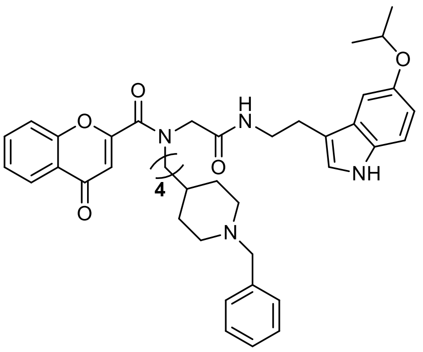

14o $C_{41}H_{48}N_4O_5$

MW: 677.370 g/mol

The crude was prepared according to ***general procedure B*** starting from *4-(1-benzylpiperidin-4-yl)butan-1-amine* **11** (246 mg, 1 mmol), paraformaldehyde **13** (30.03 mg, 1 mmol), 2-chromone-carboxylic acid **12** (190.15 mg, 1 mmol) and *3-(2-isocyanoethyl)-5-isopropoxy-1H-indole* **6** (218 mg, 1 mmol) in 10mL of CH_2Cl_2:MeOH (1:3 v/v, **0.8 mmol/mL**). After 24h, the crude was purified by flash column at 9:1 CH_2Cl_2:MeOH with NH_3 1%, to afford **14o** as a gold foam (**38 mg, 5.66%**).

^{1}H NMR (400 MHz, $CDCl_3$) δ 8.25 – 8.08 (m, 2H), 7.65 (t, J = 7.2 Hz, 1H), 7.46 (t, J = 7.4 Hz, 1H), 7.36 (d, J = 8.4 Hz, 1H), 7.34 – 7.27 (m, 4H), 7.25 – 7.15 (m, 2H), 6.99 (s, 2H), 6.81 (dd, J = 8.8, 2.0 Hz, 1H), 6.49 (s, 1H), 6.13 (t, J = 6.2 Hz, 1H), 4.50 (s, 1H), 4.00 (s, 2H), 3.73 – 3.58 (m, J = 6.1 Hz, 2H), 3.55 – 3.44 (m, 2H), 3.36 – 3.25 (m, 2H), 2.96 (t, J = 6.5 Hz, 2H), 2.90 – 2.78 (m, 2H), 1.99 – 1.80 (m, 2H), 1.68 – 1.55 (m, J = 11.8, 5.2 Hz, 3H), 1.52 (d, J = 11.0 Hz, 2H), 1.32 (d, J = 6.0 Hz, 7H), 1.14 (s, 5H).

^{13}C NMR (101 MHz, $CDCl_3$) δ 177.72, 167.71, 163.23, 155.52, 154.70, 153.02, 146.52, 136.72, 134.73, 131.77, 129.53, 128.35, 127.87, 127.23, 126.08, 124.39, 122.78, 120.00, 119.21, 118.08, 112.26, 112.18, 104.03, 71.43, 63.48, 53.78, 50.54, 39.50, 36.15, 35.60, 32.17, 29.84, 28.87, 25.04, 22.38.

HRMS ESI-TOF [M+H]+ m/z calcd. for $C_{41}H_{49}N_4O_5$: 677.3697, found: 677.3668.

***N*-(4-(1-benzylpiperidin-4-yl)butyl)-4-oxo-*N*-(2-oxo-2-((2-(5-propoxy-1H-indol-3-yl)ethyl)amino)ethyl)-4H-chromene-2-carboxamide (14p).**

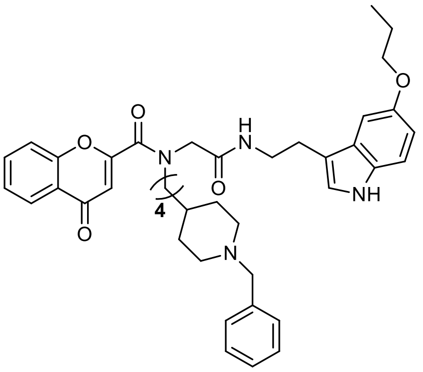

14p $C_{41}H_{48}N_4O_5$

MW: 677.370 g/mol

The crude was prepared according to ***general procedure B*** starting from *4-(1-benzylpiperidin-4-yl)butan-1-amine* **11** (250 mg, 1.05 mmol), paraformaldehyde **13** (32.91 mg, 1.05 mmol), 2-chromone-carboxylic acid **12** (208 mg, 1.05 mmol) and *3-(2-isocyanoethyl)-5-propoxy-1H-indole* **7** (270 mg, 1.05 mmol) in 10mL of CH_2Cl_2:MeOH (1:3 v/v, 0.8 mmol/mL). After 24h, the crude was purified by flash column at 9:1 CH_2Cl_2:MeOH with NH_3 1%, to afford **14p** as an orange foam **(25 mg, 3.69%)**.

^{1}H NMR (400 MHz, CDCl$_3$) δ 8.26 – 8.12 (m, 2H), 7.63 (t, J = 7.4 Hz, 1H), 7.46 (t, J = 7.4 Hz, 1H), 7.36 (d, J = 8.5 Hz, 1H), 7.33 – 7.28 (m, 5H), 7.25 – 7.14 (m, 1H), 7.00 (d, J = 13.9 Hz, 2H), 6.81 (dd, J = 8.8, 2.3 Hz, 1H), 6.48 (1, J = 92.6 Hz, 1H), 6.09 (s, 1H), 4.04 (s, 1H), 3.94 (dd, J = 13.0, 6.4 Hz, 3H), 3.64 (dd, J = 12.0, 6.1 Hz, 2H), 3.53 – 3.48 (m, 2H), 3.37 – 3.21 (m, 2H), 2.97 (t, J = 6.2 Hz, 2H), 2.90 – 2.79 (m, 2H), 1.98 – 1.74 (m, 5H), 1.60 – 1.45 (m, 5H), 1.13 (s, 5H), 1.05 (dd, J = 14.0, 6.7 Hz, 3H).

^{13}C NMR (101 MHz, CDCl$_3$) δ 177.67, 167.77, 163.23, 155.66, 153.71, 145.30, 140.17, 134.72, 131.76, 129.50, 128.34, 127.86, 127.27, 126.23, 126.09, 124.41, 123.16, 120.00, 118.21, 113.08, 112.26, 112.17, 101.82, 70.56, 63.50, 53.86, 50.53, 39.49, 36.14, 35.60, 32.15, 29.84, 28.89, 25.09, 23.84, 22.96, 10.78.

HRMS ESI-TOF [M+H]+ m/z calcd. for $C_{41}H_{49}N_4O_5$: 677.3697, found: 677.3686.

References

1. Choi JY, Calvet CM, Gunatilleke SS, et al. Rational Development of 4-Aminopyridyl-Based Inhibitors Targeting Trypanosoma cruzi CYP51 as Anti-Chagas Agents. *Journal of Medicinal Chemistry*. 2013;56(19):7651-7668. doi:10.1021/jm401067s
2. Bolea I, Juárez-Jiménez J, de los Ríos C, et al. Synthesis, Biological Evaluation, and Molecular Modeling of Donepezil and *N* -[(5-(Benzyloxy)-1-methyl-1 *H* -indol-2-yl)methyl]-*N* -methylprop-2-yn-1-amine Hybrids as New Multipotent Cholinesterase/Monoamine Oxidase Inhibitors for the Treatment of Alzheimer's Disease. *Journal of Medicinal Chemistry*. 2011;54(24):8251-8270. doi:10.1021/jm200853t
3. Benchekroun M, Bartolini M, Egea J, et al. Novel Tacrine-Grafted Ugi Adducts as Multipotent Anti-Alzheimer Drugs: A Synthetic Renewal in Tacrine-Ferulic Acid Hybrids. *ChemMedChem*. 2015;10(3):523-539. doi:10.1002/cmdc.201402409
